# The association between tea consumption and blood pressure in the adult population in Southwest China

**DOI:** 10.1186/s12889-023-15315-5

**Published:** 2023-03-13

**Authors:** Ying Zhao, Chengmeng Tang, Wenge Tang, Xuehui Zhang, Xiaoman Jiang, Zhuoma Duoji, Yixi Kangzhu, Xing Zhao, Xiaohe Xu, Feng Hong, Qiaolan Liu

**Affiliations:** 1 West China School of Public Health and West China Fourth Hospital, Sichuan University, 610041 Chengdu City, Sichuan China; 2 Chongqing Municipal Center for Disease Control and Prevention, 400042 Chongqing, China; 3 School of Public Health, Kunming Medical University, 650500 Kunming, China; 4 Chengdu Center for Disease Control and Prevention, 610041 Chengdu, China; 5 School of Medicine, Tibet University, 850000 Lhasa, China; 6 Tibet Center for disease control and prevention, 850000 Lhasa, China; 7 Department of Sociology , University of Texas at San Antonio, San Antonio, USA; 8 Department of Sociology and Psychology, School of Public Administration, Sichuan University, 610064 Chengdu, China; 9 School of Public Health, the Key Laboratory of Environmental Pollution Monitoring and Disease Control, Ministry of Education, Guizhou Medical University, Guiyang, China

**Keywords:** Hypertensive blood pressure, Systolic blood pressure, Diastolic blood pressure, Tea consumption

## Abstract

**Objectives:**

Prior research on the effect of tea consumption on blood pressure (BP) generated inconsistent findings. The objective of this study was to explore the effects of different types of tea consumption on BP.

**Methods:**

We included 76,673 participants aged 30–79 from the baseline data of the China Multi-Ethnic Cohort (CMEC) study. Binary logistic regression was used to analyze the influences of different types of tea consumption on the risk of hypertensive BP. Moreover, multiple linear regression was used to examine the association between tea drinking and BP.

**Results:**

Tea consumption was associated with a reduced risk of hypertensive BP by 10% (AOR: 0.90, 95%CI: 0.86–0.94). While dark tea was related to a 1.79–5.31 mmHg reduction in systolic blood pressure (SBP) and a 0.47–1.02 mmHg reduction in diastolic blood pressure (DBP), sweet tea, regardless of the duration, frequency, or amount of consumption, significantly was associated with a reduced SBP by 3.19–7.18 mmHg. Green tea also was associated with a reduced SBP by 1.21–2.98 mmHg. Although scented tea was related to reduced SBP by 1.26-2.48 mmHg, the greatest effect came from the long duration (> 40 years:β=-2.17 mmHg, 95%CI=-3.47 mmHg --0.87 mmHg), low frequency (1–2 d/w: β = -2.48 mmHg, 95%CI=-3.76 mmHg–-1.20 mmHg), and low amount (≤ 2 g/d: β=-2.21 mmHg, 95%CI=-3.01 mmHg–-1.40 mmHg). Additionally, scented tea was correlated to a decrease in DBP at the frequency of 1–2 d/w (β=-0.84 mmHg, 95%CI=-1.65 mmHg–-0.02 mmHg). Drinking black tea only was associated with lowered SBP. The protective effect of black tea on SBP was characterized by the long-duration (> 15 years, -2.63–-5.76 mmHg), high frequency (6–7 d/w, -2.43 mmHg), and medium amount (2.1-4.0 g/d, -3.06 mmHg).

**Conclusion:**

Tea consumption was associated with lower SBP and a reduced risk of hypertensive BP. The antihypertensive effect varies across types of tea consumed.

**Supplementary Information:**

The online version contains supplementary material available at 10.1186/s12889-023-15315-5.

## Introduction

As a common chronic non-communicable disease, hypertension has become a global challenge to public health [1]. Due to the rapid growth of the aging population, the number of people with elevated blood pressure increased by 90% from 1975 to 2015, with the majority of the increase occurring in low-income and middle-income countries or regions [2]. The prevalence of hypertension in China showed an upward trend [3], increasing from 13.6% to 1991 to 27.9% in 2015 [4]. Elevated blood pressure is significantly associated with a higher risk of cardiovascular disease and kidney-related diseases [5–8]. It has become a major cause of death and is linked to the reduction of disability-adjusted life-year[9]. However, once the diastolic blood pressure (DBP) reduces by 2 mmHg, the incidence of coronary heart disease and stroke can decrease by 6% and 15%, respectively [10]. A systematic review of 48 randomized clinical trials showed a 10% reduction in the risk of cardiovascular events for every 5 mmHg reduction in systolic blood pressure (SBP)[11].Therefore, effective prevention and control of hypertension are of crucial significance to public health.

Tea is a popular beverage worldwide, especially in Asia [12]. The association between tea consumption and incident hypertension, however, remains uncertain. Several studies have shown that drinking tea was associated with a reduced risk of hypertension, type 2 diabetes, and cardiovascular disease[13–15]. The protective effect is due primarily to the antihypertensive active ingredients, such as the tea polyphenols[16, 17]. Due to the different degrees of fermentation, the antihypertensive active ingredients in tea vary. The main antihypertensive component of green tea is catechins. In black tea, most catechins are oxidized into the thearubigins and theaflavins with weak antioxidant capacity during the fermentation process [18, 19]. In addition, the amount, duration, and frequency of tea consumption may also affect the antihypertensive effect[20–22]. On the other hand, while some studies showed that tea consumption was not associated with a reduced risk of hypertension[23–25], others even demonstrated an increased risk[26]. In addition, most of these studies focused on green tea and black tea. Rarely did they investigate the association of other types of tea consumption with blood pressure[20, 27]. The lack of population representativeness, relatively small sample size, and limited types of tea might be responsible for these inconsistent results. Therefore, it is necessary to explore the effects of different types of tea consumption and tea consumption habits on blood pressure in a large sample of the population to make scientific recommendations for lowering blood pressure.

China has a long history of tea consumption with a sizable tea-drinking population. According to the 2011 China Health and Nutrition Survey, the rates of tea drinkers in urban and rural areas were 46.5% and 33.0%, respectively [28]. As the center of the origin of tea trees, tea consumption is high in Southwest China. According to the China Patient-Centered Evaluative Assessment of Cardiac Events (PEACE) Million Persons Project, the prevalence of hypertension among adults in western China was 40.60% in 2014–2017, which was lower than the national prevalence of 44.72%[29]. But it is not clear whether tea consumption plays a role in the low prevalence of hypertension. Thus, exploring the association between tea consumption and hypertension in Southwest China can help establish such an association in the population. Moreover, Southwest China is home to multiple ethnic groups and several types of tea. In addition to green tea and black tea, dark tea, scented tea, and sweet tea are also popular, making the region an ideal location to evaluate the effects of different types of tea on blood pressure.

Based on the baseline data of the China Multi-ethnic cohort (CMEC) study, this study aimed (1) to explore the relationships between different types of tea consumption and hypertensive BP; (2) to analyze the effects of different tea consumption habits (duration of tea consumption, frequency of tea consumption, and amount of tea consumption) by different types of tea consumption on DBP and SBP; (3) to explore the modification effect of demographics and lifestyle factors on the association between tea consumption and hypertensive BP in a large population in southwest China, a low-income and middle-income region.

## Materials and methods

### Participants

**Fig. 1:**
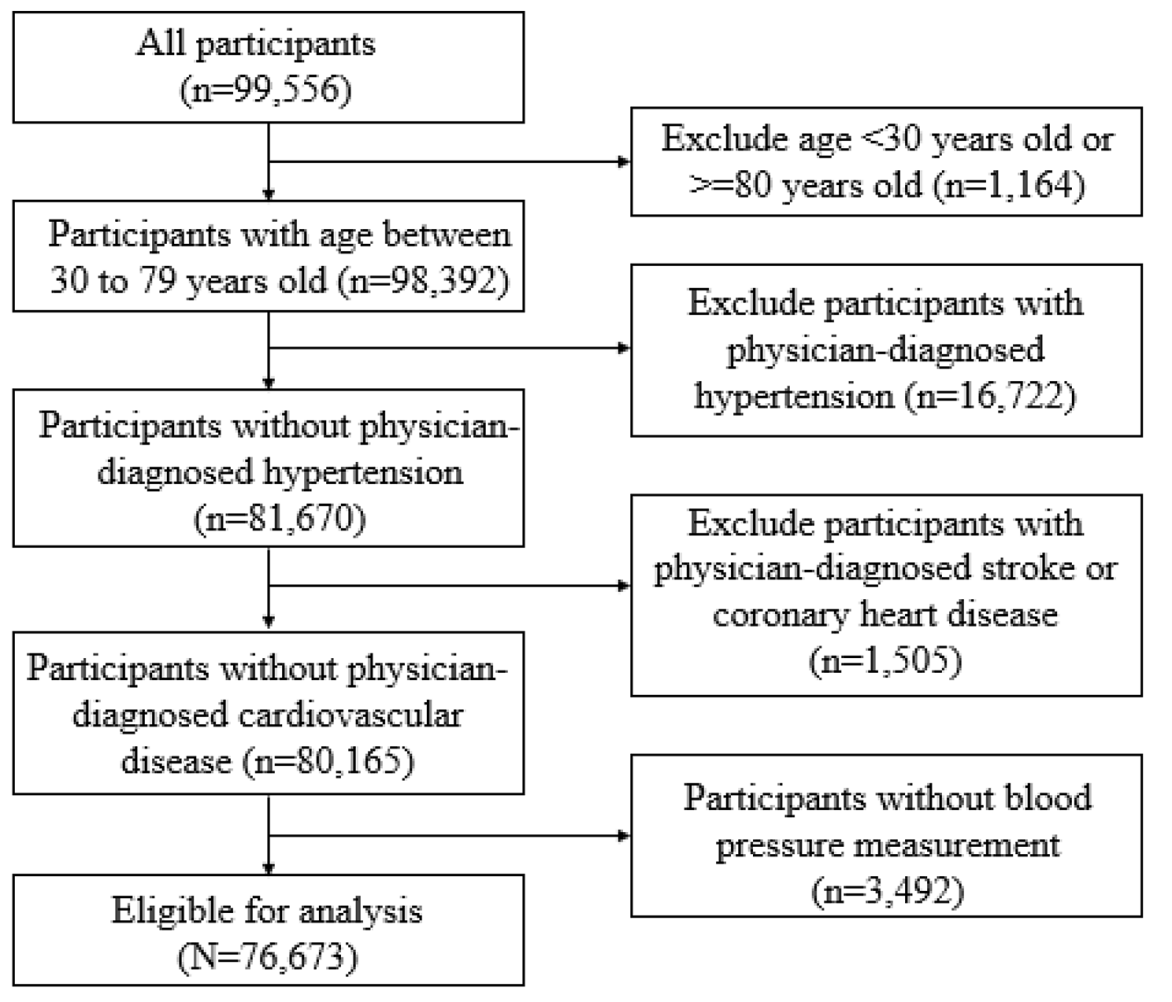
Flowchart for participants selection

A multistage, stratified cluster sampling technique was employed to conduct the China Multi-ethnic Cohort (CMEC) study in 5 provinces in Southwest, including Sichuan, Chongqing, Tibet, Guizhou, and Yunnan provinces. The baseline survey of CMEC was fielded from May 2018 to September 2019. It enrolled a total of 99,556 participants aged 30–79 (few of the Tibetan participants were younger than 30 years old). The specific investigation methods have been described elsewhere [30]. Participants were excluded from this study if they had (1) any physician-diagnosed hypertension (considering that hypertensive patients might change the habit of tea consumption), (2) any physician-diagnosed cardiovascular disease (considering that cardiovascular disease patients might change the habit of tea consumption), or (3) didn’t have three blood pressure measures. After these exclusions, 76,673 participants were included (Fig. 1). All the participants signed an informed consent form before data collection. Ethical approval for this study was obtained from the Sichuan University Medical Ethical Review Board (K2016038, K2020022).

### Data collection

The data used in this study came from the electronic questionnaire and medical examination of the CMEC baseline survey. The electronic questionnaires were conducted through face-to-face interviews, and tablet computers with a CMEC application (CMEC App) were used to record questionnaire information. The CMEC App, developed by the research team, featured an automatic recording function. The interviewers were recruited from local universities or colleges with medical backgrounds. All interviewers were trained before conducting the interviews. Through a unified and standardized process, each investigation lasted for 30–45 min. The questionnaire included sociodemographic characteristics, lifestyle questions (e.g., smoking, alcohol consumption, tea consumption, dietary habits, and physical activity), physician-diagnosed diseases, family history of diseases, reproductive information, and psychological conditions.

### Measures

#### Hypertensive blood pressure

The blood pressure was measured by an electronic sphygmomanometer, which was calibrated before use. The process of measurement followed the standard procedure of the American Heart Association[31]. Before the measurement was taken, all participants were asked to not smoke, drink (alcohol, coffee, and tea), and exercise for at least 30 min. When measuring, all participants were required to keep an upright seated position. A total of 3 SBP and DBP measures were taken and recorded. Participants who met one of the following criteria were considered as hypertensive status. The criteria were: (1) the average measured SBP of 3 times ≥ 140 mmHg and (2) the average measured DBP of 3 times ≥ 90 mmHg.

#### Tea consumption

Variables for tea consumption contained the status, type, duration, frequency, and amount of tea consumed in the past or present.

1. For the status of tea consumption, participants were asked: “Have you ever drunk tea every week for more than six months? (Yes/ No)”. Participants who answered “Yes” were queried further about other variables of tea consumption.
2. For the type of tea consumed, participants were asked: “What kind of tea do you most commonly drink?”. Responses included green tea, scented tea, dark tea, sweet tea, black tea, oolong tea, yellow tea, and white tea. The last three kinds of tea are consumed by a small number of participants, therefore, they are combined and collectively referred to as other tea in the analysis that follows. Dark tea included brick tea and Pu’er tea. Sweet tea was black tea with milk added.
3. For the duration of tea consumption, both the past drinkers and current drinkers were asked about the age when they began to drink tea. Duration of tea consumption was obtained by subtracting the age of starting drinking from the age at which the tea drinkers stopped drinking or the study time for the current drinkers. Duration of tea consumption was recoded into “no”, “≤ 15 years”, “16–40 years”, and “>40 years”.
4. The frequency of tea consumption was ascertained by one survey question: “How many days did you drink tea per week on average in the past year?” The answers were “no”, “1–2 d/w”, “3–5 d/w”, and “6–7 d/w”.
5. For the amount of tea consumption, participants were asked: “When drinking tea, how many times do you add new tea in one day?” and “How much do you usually add each time? (g/d)”. The amount of tea consumed was calculated by the equation: daily amount= (the times of adding new tea + 1)× the weight of tea added each time. The amount of tea consumption was recoded into “no”, “≤ 2.0 g/d”, “2.1-4.0 g/d”, “4.1-8.0 g/d”, and “≥ 8.1 g/d”.

#### Covariates

The covariates included age (“30–39”, “40–49”, “50–59”, “60–69”, and “70–79”), sex (“male” and “female”), ethnicity (“Han”, “Dong”, “Bouyi”, “Yi”, “Miao”, “Bai”, and “Tibetan”), marital status (“married”, “divorced”, “widowed”, and “single”), education (“illiteracy”, “primary”, “middle school”, and “college”), occupation (“employed”, “unemployed”, and “retired”), family income (“<12,000 RMB/year”, “12,000–19,999 RMB/year”, “20,000–59,999 RMB/year”, “60,000–99,999 RMB/year”, “100,000-199,999 RMB/year”, and “≥200,000 RMB/year”), BMI (“normal”, “overweight”, and “obesity” ), non-sedentary metabolic equivalent (MET) (“low”, “middle”, and “high”), smoking status (“no”, “current” and “quit”), alcohol use status (“no”, “occasionally” and “frequently”), salt intake (g/w), vegetables intake (g/w), fruits intake (g/w), dairy intake (g/w), physician-diagnosed diseases (“no” and “yes”), and family history of hypertension (“no” and “yes”). Physician-diagnosed diseases referred to whether participants had other chronic diseases or cancer, except for hypertension. The family history of hypertension was indicated by the participant’s direct relatives who had been diagnosed with hypertension by doctors.

### Data analysis

The Chi-square test was conducted for univariate analysis. The covariates that were statistically significant (*P* < 0.05) in the univariate analysis were used in the subsequent analysis as adjustment variables. The association between tea consumption and blood pressure was analyzed in two parts. In the first part, hypertensive BP was used as the dependent variable and a series of binary logistic regression models were developed to estimate the effects of the status and types of tea consumption net of statistical controls. Adjusted odds ratios (AORs) and 95% confidence intervals (CI) were reported. In the second part, multiple linear regression was used to estimate the associations between types of tea consumption and the levels of SBP and DBP.

### Stratified analysis

To examine whether demographics and lifestyle factors affect the association between tea consumption and hypertensive BP, we performed a stratified analysis, stratified by age, sex, alcohol use status, smoking status, BMI, and salt intake. In each stratified analysis, the models were controlled for all other covariates except those used for stratification. We also conducted multiple linear regression analyses on the relationships of different types of tea with change values of BP according to age, sex, alcohol use status, smoking status, BMI, and salt intake.

### Sensitivity analysis

To assess the robustness of the results, we performed a series of sensitivity analyses, including participants with physician-diagnosed cardiovascular disease and physician-diagnosed hypertension and cardiovascular disease. The association between the duration and amount of tea consumption as continuous variables and the levels of SBP and DBP were evaluated with a restricted cubic spline.

R (version 3.6.3) was utilized for all statistical analyses, and *P* < 0.05 was considered statistically significant.

## Results

### General prevalence

The sociodemographic characteristics of the participants are displayed in Table 1. Among 76,673 participants, 25,315 (33.01%) reported drinking tea, with the proportion of green tea consumption at 47.39%, dark tea consumption at 19.57%, scented tea at 17.86%, sweet tea at 9.64%, and black tea at 2.80%. Compared with non-tea drinkers, tea drinkers were more likely to be male, overweight, smokers, had a history of other diseases, and lower salt intake. Among the drinkers, the duration of tea drinking was centered around 16–40 years, 75.80% drank tea almost every day, and the amount of tea they drank was, by and large, less than 4.0 g/d. The green tea and scented tea drinkers were likely to be Han Chinese, whereas the dark tea and sweet tea drinkers were likely to be Tibetan (Table 1). The number of participants with hypertensive BP was 14,624 (19.07%), and they were more likely to be male, widowed, retired, overweight or obesity, had a lower education level and lower family income, had a lower physical activity level, and had more likely to smoke and drink alcohol. In addition, those with higher salt intake and lower fruit and dairy intake had a higher prevalence of hypertensive BP (Table S1, Supplementary material).

### The association between tea consumption and hypertensive BP

**Fig. 2:**
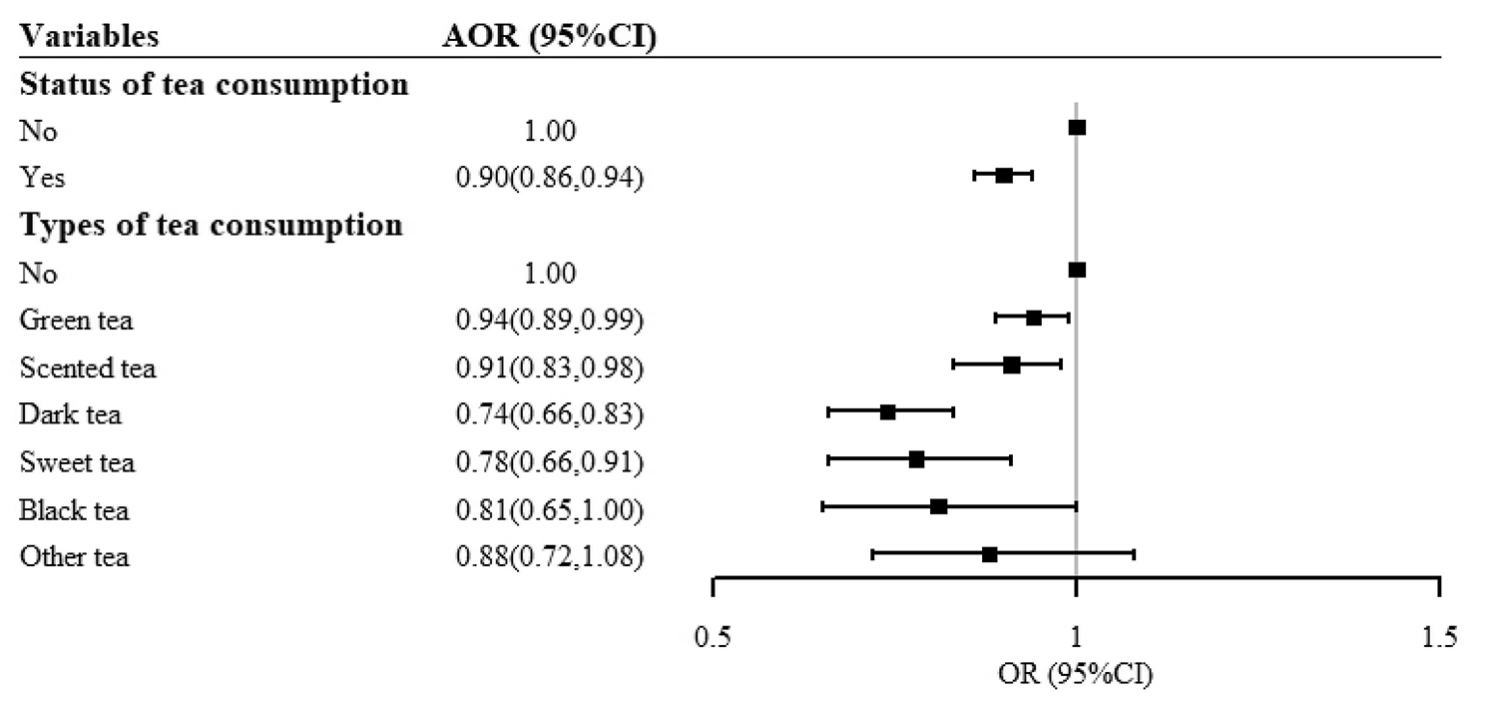
Association between type of tea consumption and hypertensive BP. (Note: AORs (95%CIs) were adjusted for age, sex, ethnicity, marital status, education, occupation, family income, BMI, MET, smoking status, alcohol use status, salt intake, vegetable intake, fruits intake, dairy intake, family history of hypertension, and physician-diagnosed diseases)

Overall, tea consumption was associated with 10% lower risk of hypertensive BP net of confounders (AOR: 0.90, 95%CI: 0.86–0.94). Compared with non-drinkers, participants who drank green tea (AOR: 0.94, 95%CI: 0.89–0.99), scented tea (AOR: 0.91, 95%CI: 0.83–0.98), dark tea (AOR: 0.74, 95%CI: 0.66–0.83), sweet tea (AOR: 0.78, 95%CI: 0.66–0.91) and black tea (AOR: 0.81,95%CI: 0.65-1.00) were associated with a lower risk of hypertensive BP (Fig. 2).

**Table 1:** Baseline characteristics of the study participants by type of tea consumption

Covariates	Type of tea consumption							Overall	*p*
	Never	Green tea	Scented tea	Dark tea	Sweet tea	Black tea	Other tea		
**Age group (years, %)**									< 0.001
30–39	11,083(21.56)	1,642(13.69)	676(15.96)	1,251(25.26)	722(29.58)	183(25.77)	152(23.17)	15,709(20.49)	
40–49	17,292(33.64)	4,268(35.58)	1,522(33.67)	1,558(31.46)	809(33.14)	304(42.82)	210(32.01)	25,963(33.86)	
50–59	12,898(25.09)	3,498(29.16)	1,204(26.64)	1,304(26.33)	623(25.52)	141(19.86)	149(22.71)	19,817(25.85)	
60–69	7,735(15.05)	1,963(16.36)	829(18.34)	676(13.65)	236(9.67)	59(8.31)	103(15.70)	11,601(15.13)	
70–79	2,389(4.65)	625(5.21)	289(6.39)	164(3.31)	51(2.09)	23(3.24)	42(6.40)	3,583(4.67)	
**Sex (%)**									< 0.001
Male	15,203(29.58)	7,642(63.70)	3,148(69.65)	2,001(40.40)	950(38.92)	403(56.76)	353(53.81)	29,700(38.74)	
Female	36,194(70.42)	4,354(36.30)	1,372(30.35)	2,952(59.60)	1,491(61.08)	307(43.24)	303(46.19)	46,973(61.26)	
**Ethnicity (%)**									< 0.001
Han	29,862(58.10)	8,581(71.53)	4,244(93.89)	841(16.98)	19(0.78)	447(62.96)	479(73.02)	44,473 (58.00)	
Dong	4,658(9.06)	567(4.73)	27(0.60)	9(0.18)	0(0.00)	47(6.62)	32(4.88)	5,340(6.96)	
Bouyi	4,092(7.96)	408(3.40)	31(0.69)	20(0.40)	1(0.04)	17(2.39)	17(2.59)	4,586(5.98)	
Yi	4,084(7.95)	646(5.39)	11(0.24)	53(1.07)	1(0.04)	23(3.24)	5(0.76)	4,823(6.29)	
Miao	3,627(7.06)	438(3.65)	20(0.44)	7(0.14)	1(0.04)	29(4.08)	6(0.91)	4,128(5.38)	
Bai	3,423(6.66)	1,259(10.50)	16(0.35)	88(1.78)	0(0.00)	69(9.72)	4(0.61)	4,859(6.34)	
Tibetan	1,651(3.21)	97(0.81)	171(3.78)	3,935(79.45)	2,419(99.10)	78(10.99)	113(17.23)	8,464 (11.04)	
**Marital status (%)**									< 0.001
Married	45,816(89.14)	10,985(91.57)	4,148(91.77)	4,404(88.92)	2,219(90.91)	641(90.28)	581(88.57)	68,794 (89.73)	
Divorce	2,204(4.29)	410(3.42)	213(4.71)	202(4.08)	51(2.09)	39(5.49)	39(5.95)	3,158(4.12)	
Widowed	2,794(5.44)	473(3.94)	119(2.63)	191(3.86)	82(3.36)	10(1.41)	23(3.51)	3,692(4.82)	
Single	582(1.13)	128(1.07)	40(0.88)	156(3.15)	89(3.65)	20(2.82)	13(1.98)	1,028(1.34)	
**Education (%)**									< 0.001
Illiteracy	12,627(24.57)	1,919(16.00)	403(8.92)	2,792(56.37)	1,305(53.46)	102(14.37)	104(15.85)	19,252 (25.11)	
Primary	12,584(24.48)	3,079(25.67)	1,039(22.99)	1,229(24.81)	807(33.06)	115(16.20)	146(22.26)	18,999 (24.78)	
Middle school	19,917(38.75)	5,305(44.23)	2,475(54.76)	689(13.91)	299(12.25)	271(38.17)	281(42.84)	29,237 (38.13)	
College	6,269(12.20)	1,692(14.11)	603(13.34)	243(4.91)	30(1.23)	222(31.27)	125(19.05)	9,184 (11.98)	
**Occupation (%)**									< 0.001
Employed	45,125(87.88)	10,351(86.35)	3,442(76.22)	4,597(92.87)	2,363(96.80)	618(87.04)	545(83.21)	67,041 (87.51)	
Unemployed	2,351(4.58)	475(3.96)	382(8.46)	119(2.40)	29(1.19)	43(6.06)	35(5.34)	3,434(4.48)	
Retired	3,875(7.55)	1,161(9.69)	692(15.32)	234(4.73)	49(2.01)	49(6.90)	75(11.45)	6,135(8.01)	
**Family income (RMB /year) (%)**									< 0.001
< 12,000	8,744(17.03)	1,787(14.93)	419(9.28)	782(15.79)	507(20.77)	69(9.75)	75(11.45)	12,383 (16.17)	
12,000–19,999	9,182(17.89)	1,895(15.83)	544(12.05)	1,327(26.80)	809(33.14)	80(11.30)	108(16.49)	13,945 (18.21)	
20,000–59,999	18,592(36.22)	4,494(37.54)	1,762(39.04)	2,020(40.80)	819(33.55)	199(28.11)	220(33.59)	28,106 (36.70)	
60,000–99,999	7,705(15.01)	1,867(15.59)	973(21.56)	411(8.30)	189(7.74)	128(18.08)	109(16.64)	11,382 (14.86)	
100,000–199,999	5,793(11.28)	1,480(12.36)	648(14.36)	296(5.98)	86(3.52)	158(22.32)	109(16.64)	8,570 (11.19)	
>=200,000	1,321(2.57)	449(3.75)	167(3.70)	115(2.32)	31(1.27)	74(10.45)	34(5.19)	2,191(2.86)	
**BMI(kg/m2) (%)**									< 0.001
Normal	28,522(55.58)	6,584(54.95)	2,094(46.37)	1,960(39.68)	623(25.60)	353(49.72)	250(38.23)	40,386 (52.76)	
Overweight	17,414(33.93)	4,195(35.01)	1,846(40.88)	1,954(39.56)	1,464(60.15)	274(38.59)	302(46.18)	27,449 (35.86)	
Obesity	5,382(10.49)	1,202(10.03)	576(12.75)	1,025(20.75)	347(14.26)	83(11.69)	102(15.60)	8,717 (11.39)	
**MET (%)**									< 0.001
Low	11,635(22.74)	2,647(22.18)	1,548(34.42)	1,918(38.84)	889(36.64)	170(24.08)	211(32.26)	19,018 (24.92)	
Middle	26,075(50.96)	6,078(50.93)	2,159(48.00)	2,044(41.39)	1,135(46.78)	399(56.52)	329(50.31)	38,219 (50.08)	
High	13,453(26.29)	3,210(26.90)	791(17.59)	976(19.77)	402(16.57)	137(19.41)	114(17.43)	19,083 (25.00)	
**Smoking status (%)**									< 0.001
No	42,830(83.33)	6,410(53.43)	2,037(45.07)	4,040(81.57)	1,808(74.07)	439(61.83)	438(66.77)	58,002 (75.65)	
Current	6,969(13.56)	4,710(39.26)	2,020(44.69)	670(13.53)	530(21.71)	229(32.25)	176(26.83)	15,304 (19.96)	
Quit	1,598(3.11)	876(7.30)	463(10.24)	243(4.91)	103(4.22)	42(5.92)	42(6.40)	3,367(4.39)	
**Alcohol use status (%)**									< 0.001
No	30,472(59.29)	5,167(43.07)	1,628(36.02)	3,832(77.37)	1,632(66.86)	275(38.73)	266(40.55)	43,272 (56.44)	
Occasionally	16,253(31.62)	3,953(32.95)	1,524(33.72)	793(16.01)	661(27.08)	302(42.54)	260(39.63)	23,746 (30.97)	
Frequently	4,672(9.09)	2,876(23.97)	1,368(30.27)	328(6.62)	148(6.06)	133(18.73)	130(19.82)	9,655 (12.59)	
**Salt (g/w, mean ± SD)**	47.52 ± 28.64	45.91 ± 27.52	42.74 ± 24.41	43.32 ± 33.08	43.52 ± 33.62	42.28 ± 26.56	42.71 ± 28.20	46.50 ± 28.74	< 0.001
**Vegetables (g/w, mean ± SD)**	2,227.71 ± 1,416.93	2,197.89 ± 1,433.73	2,401.19 ± 1,516.65	1,655.93 ± 1,459.11	1,146.90 ± 1,366.48	2,189.56 ± 1,522.61	2,319.35 ± 1,720.95	2,162.39 ± 1,450.08	< 0.001
**Fruits (g/w, mean ± SD)**	854.48 ± 799.88	913.66 ± 813.56	894.90 ± 811.07	650.16 ± 729.30	575.48 ± 877.84	967.34 ± 879.90	894.91 ± 882.93	845.42 ± 806.07	< 0.001
**Dairy (g/w, mean ± SD)**	367.18 ± 574.19	368.40 ± 577.94	605.88 ± 691.37	477.99 ± 616.77	276.34 ± 423.35	553.02 ± 664.07	501.68 ± 647.71	388.64 ± 586.38	< 0.001
**Family history of hypertension (%)**									< 0.001
No	39,523(76.9)	9,090(75.78)	3,394(75.09)	4,200(84.80)	1,986(81.36)	519(73.10)	512(78.05)	59,224(77.24)	
Yes	11,874(23.1)	2,906(24.22)	1,126(24.91)	753(15.20)	455(18.64)	191(26.90)	144(21.95)	17,449 (22.76)	
**Physician-diagnosed disease (%)**									< 0.001
No	43,100 (83.86)	9,681(80.70)	3,443(76.17)	3,975(80.25)	1,861(76.24)	561(79.01)	482(73.48)	63,103(82.30)	
Yes	8,297(16.14)	2,315(19.30)	1,077(23.83)	978(19.75)	580(23.76)	149(20.99)	174(26.52)	13,570 (17.70)	
**Duration of tea consumption (years) (%)**									< 0.001
≤ 15	-	3,485(29.06)	1,353(29.94)	523(10.56)	139(5.69)	325(45.77)	320(48.78)	6,164(24.35)	
16–40	-	6,782(56.55)	2,601(57.56)	2,724(55.00)	1,268(51.95)	304(42.82)	246(37.50)	13,940 (55.08)	
> 40	-	1,725(14.38)	565(12.50)	1,706(34.44)	1,034(42.36)	81(11.41)	90(13.72)	5,206(20.57)	
**Frequency of tea consumption (d/w)** (**%)**									< 0.001
1–2	-	1,465(12.49)	550(12.66)	312(6.39)	77(3.16)	114(16.38)	109(17.33)	2,631(10.63)	
3–5	-	1,784(15.22)	848(19.52)	372(7.62)	97(3.99)	133(19.11)	120(19.08)	3,360(13.57)	
6–7	-	8,476(72.29)	2,947(67.83)	4,199(85.99)	2,260(92.85)	449(64.51)	400(63.59)	18,761 (75.80)	
**Amount of tea consumption (g/d) (%)**									< 0.001
≤ 2.0	-	2,713(22.75)	1,453(32.16)	909(18.36)	1,204(49.32)	147(20.85)	194(29.66)	6,621(26.25)	
2.1-4.0	-	3,708(31.09)	1,306(28.91)	899(18.16)	462(18.93)	210(29.79)	176(26.91)	6,768(26.83)	
4.1-8.0	-	3,020(25.32)	966(21.38)	1,310(26.46)	256(10.49)	149(21.13)	133(20.34)	5,847(23.18)	
≥ 8.1	-	2,486(20.84)	793(17.55)	1,832(37.01)	519(21.26)	199(28.23)	151(23.09)	5,989(23.74)	
**Hypertensive BP (%)**									< 0.001
No	41,714(81.16)	9,374(78.14)	3,502(77.48)	4,230(85.40)	2,109(86.40)	598(84.23)	522(79.57)	62,049(80.93)	
Yes	9,683(18.84)	2,622(21.86)	1,018(22.52)	723(14.60)	332(13.60)	112(15.77)	134(20.43)	14,624 (19.07)	

### The association between tea consumption and blood pressure

Compared with never drinkers, drinking green tea, scented tea, dark tea, sweet tea, and black tea was associated with a reduced the average level of SBP, whereas drinking scented tea and dark tea significantly was related to a reduced the average level of DBP. But the reduction in DBP was not observed for tea drinkers who consumed green tea, sweet tea, or black tea (Table 2).

Drinking green tea was correlated with reduced SBP, and the degree of reduction varied by the duration, frequency, and amount of tea consumption. The longer duration of tea drinking was statistically associated with the greater reduction in SBP. For example, tea consumption for more than 40 years was associated with a 2.98 mmHg (95%CI=-3.74 mmHg–-2.21 mmHg) reduction in SBP. In terms of frequency, low and high frequency tea consumption groups were correlated with a 1.35 mmHg (95%CI=-2.15 mmHg–-0.55 mmHg) and 1.76 mmHg (95%CI=-2.14 mmHg–-1.39 mmHg) decrease in SBP, respectively. In the low-dose group, the antihypertensive effect of green tea was the best, and the weakened effect of antihypertensive was associated with the increased amount of tea consumption. The reduction in SBP was in the range of 1.21–1.85 mmHg. Unlike other types of tea, green tea showed to be associated with a mild increase in DBP. However, these effects were only observed in short-middle-term drinking(≤ 40 years), medium and high-frequency drinking(> 2d/w), and medium and high amount drinking(≥ 4.1 g/d). The increase in blood pressure was small, no more than 0.7 mmHg (Table 2).

Drinking scented tea was associated with a 1.26-2.48 mmHg reduction in SBP significantly, and the greatest antihypertensive effect appeared in the low-frequency tea consumption group. That is, when the frequency of tea consumption was 1–2 d/w, and the amount was less than 2.0 g/d, it was associated with the greatest decrease in SBP, by 2.48 mmHg (95%CI=-3.76 mmHg–-1.20 mmHg) and 2.21 mmHg (95%CI=-3.01 mmHg–-1.40 mmHg), respectively. The longer duration of scented tea consumption was associated a more significant reduction in SBP. For instance, the duration of tea consumption more than 40 years was associated with a reduction in SBP of 2.17 mmHg (95%CI=-3.47 mmHg --0.87 mmHg). The effect of scented tea on DBP was only observed in those who consumed scented tea 1–2 days per week (β=-0.84 mmHg, 95%CI=-1.65 mmHg–-0.02 mmHg) (Table 2).

Regardless of the duration, dark tea consumption was linked to a reduced SBP by a range of 1.79–4.19 mmHg. When dark tea consumption was 1-2d/w and 6-7d/w, it was associated with a 2.41 mmHg (95%CI=-4.13 mmHg–-0.70mmHg) and 2.90 mmHg (95%CI=-3.58 mmHg–-2.21 mmHg) reduction in SBP, respectively, whereas it was related to a 0.47 mmHg (95%CI=-0.91 mmHg–-0.03 mmHg) decrease in DBP when dark tea consumption was and 6-7 d/w. When drinking ≤ 2.0 g of dark tea per day, it was correlated the best antihypertensive effect. The amount of reduction in SBP was 5.31 mmHg (95%CI=-6.41 mmHg–-4.20 mmHg) (Table 2).

Sweet tea, regardless of the duration, frequency, or amount consumed, was correlated to a reduced SBP by a range of 3.19–7.18 mmHg. When drinking sweet tea for more than 40 years, the frequency was 3-5d/w and the amount was 2.1-4.0 g per day, it was linked to a 5.64 mmHg (95%CI=-6.83 mmHg–-4.45 mmHg), 6.26mmHg (95%CI=-9.34 mmHg–-3.18 mmHg), and 7.18 mmHg (95%CI=-8.74 mmHg–-5.62 mmHg) decrease in SBP, respectively. However, when drinking sweet tea for more than 40 years and the amount of tea consumed was ≤ 2.0 g/d, sweet tea was connected with a slight DBP boosting effect: an increase in DBP by 1.60 mmHg and 1.38 mmHg, respectively (Table 2).

Drinking black tea was only associated with lowered SBP. Long-duration, high-frequency, and medium-amount drinking groups were associated with a significant reduction in SBP. When drinking black tea for more than 15 years, and the frequency was 6–7 d/w, it was connected with an SBP reduction of 2.43-5.76 mmHg. Moreover, drinking black tea less than 8.0 g/d was associated with an SBP reduction of 2.67-3.06 mmHg (Table 2).

**Table 2:** The association between type of tea consumption and SBP/DBP

	Green tea		Scented tea		Dark tea		Sweet tea		Black tea
	β(95%*CI*)		β(95%*CI*)		β(95%*CI*)		β(95%*CI*)		β(95%*CI*)
***Outcome = SBP***									
**Duration of tea consumption (years)**									
≤ 15	-0.24(-0.77,0.30)		-1.42(-2.25,-0.59)		-2.66(-3.99,-1.33)		-5.35(-7.96,-2.73)		-0.65(-2.31,1.02)
16–40	-1.88(-2.29,-1.47)		-1.88(-2.51,-1.25)		-1.79(-2.55,-1.02)		-5.08(-6.20,-3.97)		-2.63(-4.36,-0.91)
> 40	-2.98(-3.74,-2.21)		-2.17(-3.47,-0.87)		-4.19(-5.12,-3.26)		-5.64(-6.83,-4.45)		-5.76(-9.07,-2.45)
**Frequency of tea consumption (d/w)**									
1–2	-1.35(-2.15,-0.55)		-2.48(-3.76,-1.20)		-2.41(-4.13,-0.70)		-5.18(-8.64,-1.71)		-0.59(-3.39,2.20)
3–5	-0.70(-1.43,0.04)		-1.26(-2.30,-0.23)		-0.33(-1.92,1.27)		-6.26(-9.34,-3.18)		-2.14(-4.73,0.44)
6–7	-1.76(-2.14,-1.39)		-1.82(-2.41,-1.22)		-2.90(-3.58,-2.21)		-5.30(-6.26,-4.33)		-2.43(-3.86,-1.01)
**Amount of tea consumption (g/d)**									
≤ 2.0	-1.85(-2.44,-1.25)		-2.21(-3.01,-1.40)		-5.31(-6.41,-4.20)		-5.29(-6.42,-4.15)		-2.72(-5.17,-0.26)
2.1-4.0	-1.67(-2.19,-1.15)		-1.60(-2.44,-0.75)		-4.61(-5.69,-3.53)		-7.18(-8.74,-5.62)		-3.06(-5.13,-0.99)
4.1-8.0	-1.32(-1.90,-0.74)		-2.14(-3.13,-1.16)		-0.29(-1.26,0.67)		-6.61(-8.60,-4.62)		-2.67(-5.13,-0.22)
≥ 8.1	-1.21(-1.85,-0.57)		-0.72(-1.80,0.37)		-1.81(-2.67,-0.96)		-3.19(-4.69,-1.69)		-0.22(-2.35,1.91)
***Outcome = DBP***									
**Duration of tea consumption (years)**									
≤ 15	0.55(0.21,0.89)		0.31(-0.21,0.84)		-0.76(-1.61,0.09)		1.41(-0.26,3.08)		-0.25(-1.31,0.81)
16–40	0.31(0.05,0.58)		0.08(-0.33,0.48)		-0.96(-1.45,-0.47)		-0.53(-1.25,0.18)		-0.54(-1.64,0.56)
> 40	0.30(-0.18,0.79)		-0.14(-0.97,0.69)		0.45(-0.14,1.05)		1.60(0.84,2.36)		-0.62(-2.73,1.49)
**Frequency of tea consumption (d/w)**									
1–2	0.08(-0.43,0.59)		-0.84(-1.65,-0.02)		-0.43(-1.52,0.67)		-0.18(-2.39,2.04)		-1.19(-2.98,0.59)
3–5	0.53(0.07,1.00)		0.16(-0.50,0.82)		-1.02(-2.04,0.00)		-0.46(-2.43,1.51)		-0.09(-1.74,1.55)
6–7	0.44(0.20,0.68)		0.37(-0.01,0.75)		-0.47(-0.91,-0.03)		0.55(-0.07,1.16)		-0.24(-1.15,0.66)
**Amount of tea consumption (g/d)**									
≤ 2.0	0.33(-0.05,0.71)		0.05(-0.46,0.56)		0.31(-0.40,1.02)		1.38(0.65,2.11)		-1.20(-2.76,0.37)
2.1-4.0	0.11(-0.23,0.44)		0.13(-0.41,0.67)		-1.02(-1.72,-0.33)		-0.76(-1.75,0.24)		-0.73(-2.04,0.59)
4.1-8.0	0.65(0.29,1.02)		-0.06(-0.69,0.56)		-0.26(-0.88,0.35)		-0.45(-1.72,0.82)		0.11(-1.45,1.67)
≥ 8.1	0.51(0.10,0.92)		0.56(-0.13,1.26)		-0.86(-1.41,-0.31)		0.06(-0.90,1.02)		0.26(-1.09,1.62)

Note: Adjusted for age, sex, ethnicity, marital status, education, occupation, family income, BMI, MET, smoking status, alcohol use status, salt intake, vegetable intake, fruits intake, dairy intake, family history of hypertension, and physician-diagnosed diseases. β indicates unstandardized partial regression coefficient

**Fig. 3:**
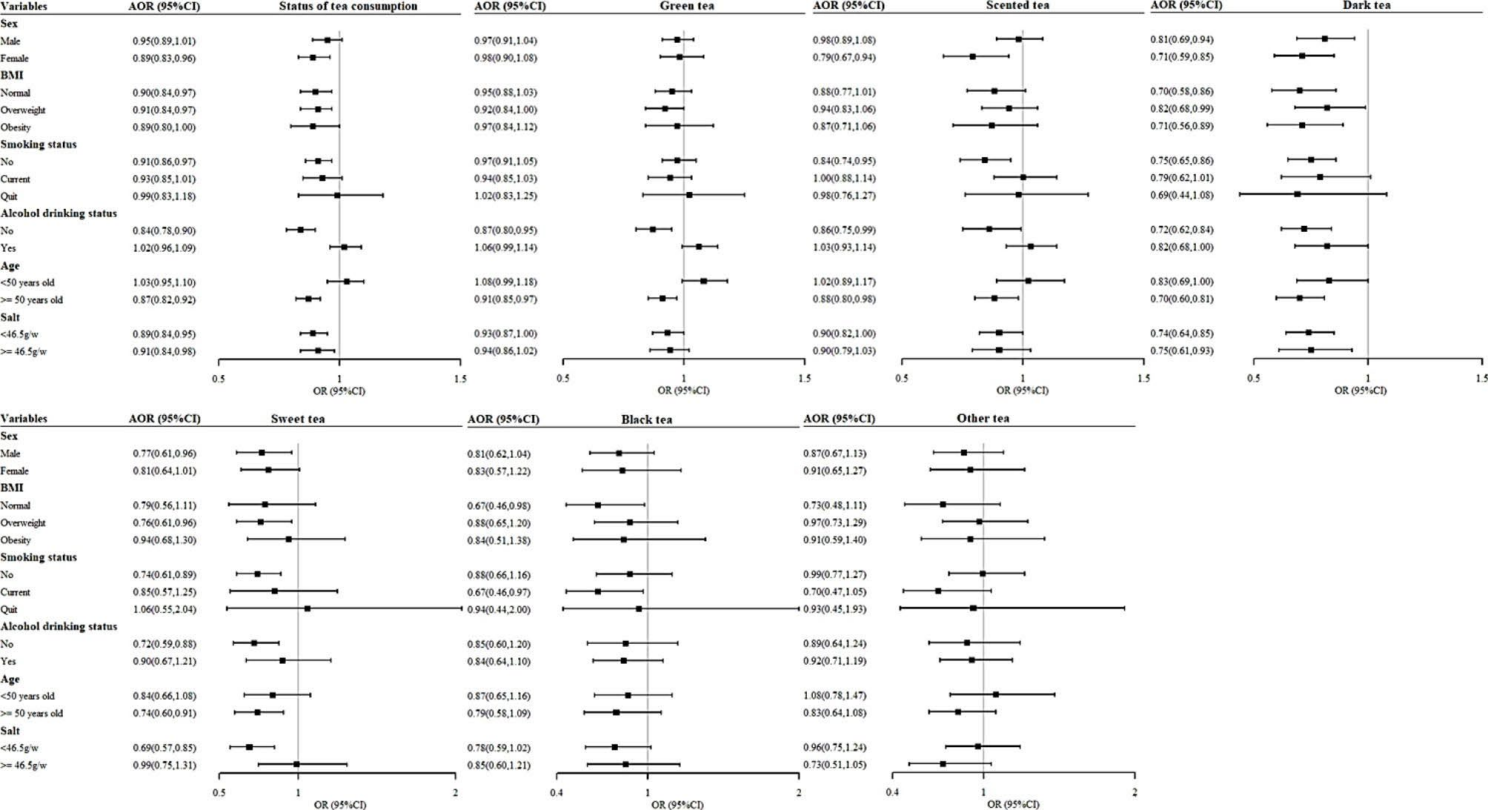
Associations between different types of tea consumption and hypertensive BP according to sex, BMI, smoking status, alcohol use status, age, and salt intake. (Note: AORs (95%CIs) were adjusted for age, sex, ethnicity, marital status, education, occupation, family income, BMI, MET, smoking status, alcohol use status, salt intake, vegetable intake, fruits intake, dairy intake, family history of hypertension, and physician-diagnosed diseases)

### Stratified analysis

Stratified analyses suggested that the association between the status of tea consumption and hypertensive BP was modified by sex, BMI, smoking status, alcohol use status, age, and salt consumption. That is, compared with those who never drank tea, tea consumption was associated with a lowered risk of hypertensive BP for women (AOR: 0.89, 95%CI: 0.83–0.96), those with normal-weight (AOR: 0.90, 95%CI: 0.84–0.97) or overweight (AOR: 0.91, 95%CI: 0.84–0.97), those who never smoked (AOR: 0.91, 95%CI: 0.86–0.97) or never drank alcohol (AOR: 0.84, 95%CI: 0.78–0.90), and those who were over 50 years old of age (AOR: 0.87, 95%CI: 0.82–0.92). Among participants with above-average salt intake, the protective effect of tea consumption on hypertensive BP was weakened. The associations between different types of tea consumption and hypertensive BP were also modified by sex, BMI, smoking status, alcohol use status, age, and salt consumption (Fig. 3). The relationships between consuming behaviors of different types of tea and BP stratified by sex, BMI, smoking status, alcohol drinking status, age, and salt was shown in Table S2 (Supplementary material).

### Sensitivity analysis

The results of the analyses for participants with physician-diagnosed cardiovascular disease were consistent with those reported above. Green tea, scented tea, dark tea, sweet tea, and black tea all linked to lower risk of hypertensive BP (Fig S1, Supplementary material). Similarly, the results of the analyses for participants with physician-diagnosed hypertension and cardiovascular disease also showed tea consumption was associated with a protective effect on hypertensive BP (Fig S2, Supplementary material). Finally, the results of restricted cubic spline analyses indicated that the gradual decrease in SBP correlated with the increase in drinking duration of green tea, scented tea, dark tea, sweet tea, and black tea, and an L-shaped or U-shaped pattern was observed between SBP and the amount of tea consumed (Fig S3-7, Supplementary material).

## Discussion

It is the first study on the association between tea consumption and blood pressure in southwest China. In this large community-based study, we found statistically significant and net associations between different types of tea consumption (green tea, scented tea, dark tea, sweet tea, and black tea) and reduced SBP in the adult population (aged 30–79) in Southwest China. Furthermore, we found that dark tea consumption was significantly associated with a lower DBP in the same study population. In the pages that follow, we reiterate these findings in detail.

Our study found that tea consumption was associated with a reduced risk of hypertensive BP, which was consistent with the majority of previous research findings[27, 32–34]. For example, a meta-analysis of 25 randomized controlled trials showed that habitual tea consumption was significantly associated with reduced blood pressure[22]. A large cohort study in China also reported that habitual tea consumption was associated with a decreased risk for incident hypertension (by 14% with HR = 0.86, 95%CI: 0.80–0.91) and a lowered risk for blood pressure progression (by 17% with OR = 0.83, 95%CI: 0.79–0.88). These findings suggest that not only did habitual tea drinking was associated with the reduced risk of hypertensive BP but also provided a preventive effect against blood pressure progression[35]. Another study of 4,579 elderly people in Jiangsu province of China found that habitual tea consumption was negatively associated with the prevalence of hypertension and SBP level[32]. On the other hand, a cohort study of Iranian adults found that tea consumption was not associated with a lowered risk of hypertension after six years of follow-up[24]. A more inconsistent finding came from a Chinese cohort study of 59,693 subjects, which found that habitual tea consumption was associated with a slightly higher risk of hypertension after 7.1 years of follow-up[26]. The reasons for these inconsistent results may be related to differences in the study population and measurement methods of tea consumption.

This study found that green tea consumption was associated with a 6% lower risk of hypertensive BP. Green tea has the best SBP-lowering associated effect when the consumption is long-term, low in amount, and less frequent. Green tea is unfermented and contains more catechins than other teas. Previous studies have shown that catechins can improve vascular endothelial function by promoting NO production and enhancing NO bioavailability, thereby reducing blood pressure[36]. Green tea catechins also protect blood vessels by inhibiting angiotensinase production[37]. Like previous study findings[26], this study also generated an inconsistent result. That is, green tea was associated with a slight increase in DBP (no more than 0.7mmHg) with short duration, medium or high frequency, and medium or high amount of consumption. This unexpected finding might be related to the caffeine contained in green tea.

Drinking dark tea was associated with a reduced risk of hypertensive BP by 26%. In effect, dark tea consumption was associated with a significantly reduction in both SBP and DBP, which was not found in other teas, except that scented tea correlated to slightly reduced the DBP in the low-frequency group. Dark tea belongs to post-fermented tea. During the fermentation process, catechins are converted into the theabrownie, which leads to changes in its biological activity [38]. Previous studies found that dark tea had the effects of improving hyperlipidemia and reducing the risk of diabetes [39, 40]. There are two explanations for why dark tea consumption can reduce BP. First, dark tea had stronger antioxidant properties and could protect the vascular endothelium from damage by reactive oxygen species and free radicals [41, 42]. Second, unlike other teas, the blood pressure reduction mechanism of dark tea does not depend on the vascular endothelium. Instead, it inhibites Ca^2 +^ influx to reduce vasoconstriction by blocking voltage-dependent calcium channels [43].

When the dosage of tea consumption was in the range of 2.1-4.0 g/d, sweet tea associated with a reduced SBP by 7.18mmHg. Paradoxically, sweet tea was associated with a slight increase in DBP when the consumption endured for more than 40 years and the amount was less than 2.0 g per day. The effect of sweet tea to increase DBP might be related to its special preparation method. Sweet tea is made by adding milk to black tea, which may partially explain a slight increase in DBP. Previous studies found that adding milk to black tea increased both SBP and DSP, but the exact mechanism was unclear[44].

Scented tea was also associated with lower DBP, but only at the low frequency of consumption. Scented tea is made from dried flowers and green tea. Scented tea contains chlorogenic acid and anthocyanins [45]. Laboratory evidence indicates that anthocyanins can induce eNOS expression in vascular endothelial cells through the Src-ERK1/2-Sp1 signaling pathway, promote NO production [46], inhibit the activity of the angiotensin-converting enzyme [47], and regulate aldosterone activity, thus producing a hypotensive effect [48]. As natural phytochemicals, the polyphenols and flavonoids in tea has additive and synergistic antioxidant activities [49], and the combined health effects were greater than that of a single substance. Therefore, the antihypertensive effect of scented tea might be better than that of single tea.

Our stratified analysis showed that the association between tea consumption and the risk of hypertensive BP was susceptible to demographic and lifestyle influences. Consistent with Tong et al.’s study, which found that green tea consumption was inversely correlated with five-year blood pressure changes in Chinese adults, but smoking attenuated the effect[50], our study indicated that tea consumption was associated with a reduced risk of hypertensive BP in nonsmokers, but not in smokers. In addition, our study results showed that tea drinking was associated with a further reduction in the risk of hypertensive BP if healthy lifestyles were practiced, such as no smoking or no drinking. It is well established that unhealthy lifestyle behaviors such as smoking, drinking alcohol, or a high-salt diet were related to a higher risk of high blood pressure, thereby attenuating the protective effect of tea consumption on blood pressure.

## Strengths and limitations

There are several strengths and limitations in this study. First, the large sample, multi-ethnic natural cohort study has a better representation of the population, which increased the credibility and generalizability of the study findings. Second, this study explored the antihypertensive effects of five types of tea consumption on SBP and DBP, including green tea, dark tea, scented tea, sweet tea, and black tea. The detailed analyses of the associations between frequency, duration, and amount of tea consumption and blood pressure were adjusted for various confounders, which allowed us to offer specific recommendations about tea consumption. Third, stratified and sensitivity analyses were conducted to explore the association between tea consumption and hypertensive BP by different sociodemographic characteristics and lifestyle variations, which made the results more robust and generalizable.

There are several study limitations as well. First, this study might suffer from an unspecified amount of recall bias associated with self-reported data. However, our carefully designed research protocol, such as the questionnaire development and interviewing technique might have helped us to minimize this possible bias. Second, the information about the tea brewing method was not collected and analyzed. Different brewing methods entail differences in time and temperature, which could have changed the bioactive ingredient of tea to affect the association between tea consumption and blood pressure [19]. Third, the content of the substance in tea was not measured in this study, as such, the confounding effect of caffeine was not identified and excluded. Finally, this was a cross-sectional study, which would not allow us to establish a causal link between tea consumption and blood pressure. Such a causal relationship can be confirmed by randomized controlled trials or panel studies in the future.

## Conclusion

Tea consumption is associated with a protective effect on blood pressure by lowering the risk of hypertensive BP by 10%. However, the protective effects vary across the type of tea consumed. Dark tea is related to lower SBP irrespective of duration and frequency of consumption. Long duration of green tea, scented tea, black tea, and sweet tea consumption is associated with decreased SBP, but the antihypertension effects vary in frequency, amount, and types of tea consumed.

## Electronic supplementary material

Below is the link to the electronic supplementary material.

Supplementary Material 1

## Data Availability

The data and materials used in this paper are not public without the permission of Sichuan University. If there is a reasonable request, please contact the corresponding author for more data.

## References

[1] Mills KT, Stefanescu A, He J (2020). The global epidemiology of hypertension. Nat Rev Nephrol.

[2] Collaboration NCDRF (2017). Worldwide trends in blood pressure from 1975 to 2015: a pooled analysis of 1479 population-based measurement studies with 19.1 million participants. Lancet.

[3] Wang Z, Chen Z, Zhang L, Wang X, Hao G, Zhang Z, Shao L, Tian Y, Dong Y, Zheng C, Wang J, Zhu M, Weintraub WS, Gao R (2018). Status of hypertension in China: results from the China Hypertension Survey, 2012–2015. Circulation.

[4] Luo Y, Xia F, Yu X, Li P, Huang W, Zhang W (2021). Long-term trends and regional variations of hypertension incidence in China: a prospective cohort study from the China Health and Nutrition Survey, 1991–2015. BMJ Open.

[5] Global Burden of Metabolic Risk Factors for Chronic Diseases C (2014). Cardiovascular disease, chronic kidney disease, and diabetes mortality burden of cardiometabolic risk factors from 1980 to 2010: a comparative risk assessment. Lancet Diabetes Endocrinol.

[6] Moghani Lankarani M, Assari S (2017). Diabetes, hypertension, obesity, and long-term risk of renal disease mortality: racial and socioeconomic differences. J Diabetes Investig.

[7] Di Palo KE, Barone NJ (2020). Hypertension and heart failure: Prevention, targets, and treatment. Heart Fail Clin.

[8] Cipolla MJ, Liebeskind DS, Chan SL (2018). The importance of comorbidities in ischemic stroke: impact of hypertension on the cerebral circulation. J Cereb Blood Flow Metab.

[9] Zhou M, Wang H, Zeng X, Yin P, Zhu J, Chen W, Li X, Wang L, Wang L, Liu Y, Liu J, Zhang M, Qi J, Yu S, Afshin A, Gakidou E, Glenn S, Krish VS, Miller-Petrie MK, Mountjoy-Venning WC, Mullany EC, Redford SB, Liu H, Naghavi M, Hay SI, Wang L, Murray CJL, Liang X (2019). Mortality, morbidity, and risk factors in China and its provinces, 1990–2017: a systematic analysis for the global burden of Disease Study 2017. Lancet (London England).

[10] Cook NR, Cohen J, Hebert PR, Taylor JO, Hennekens CH (1995). Implications of small reductions in diastolic blood pressure for primary prevention. Arch Intern Med.

[11] Canoy D, Nazarzadeh M, Copland E, Bidel Z, Rao S, Li Y, Rahimi K (2022). How much lowering of blood pressure is required to prevent Cardiovascular Disease in patients with and without previous Cardiovascular Disease?. Curr Cardiol Rep.

[12] Weisburger JH (1997). Tea and health: a historical perspective. Cancer Lett.

[13] Yang YC, Lu FH, Wu JS, Wu CH, Chang CJ (2004). The protective effect of habitual tea consumption on hypertension. Arch Intern Med.

[14] Nie J, Yu C, Guo Y, Pei P, Chen L, Pang Y, Du H, Yang L, Chen Y, Yan S, Chen J, Chen Z, Lv J, Li L (2021). Tea consumption and long-term risk of type 2 diabetes and diabetic complications: a cohort study of 0.5 million chinese adults. Am J Clin Nutr.

[15] Tian T, Lv J, Jin G, Yu C, Guo Y, Bian Z, Yang L, Chen Y, Shen H, Chen Z, Hu Z, Li L, China Kadoorie Biobank Collaborative G (2020). Tea consumption and risk of stroke in chinese adults: a prospective cohort study of 0.5 million men and women. Am J Clin Nutr.

[16] Hugel HM, Jackson N, May B, Zhang AL, Xue CC (2016). Polyphenol protection and treatment of hypertension. Phytomedicine.

[17] Giglio RV, Patti AM, Cicero AFG, Lippi G, Rizzo M, Toth PP, Banach M (2018). Polyphenols: potential use in the Prevention and Treatment of Cardiovascular Diseases. Curr Pharm Design.

[18] Lorenz M, Urban J, Engelhardt U, Baumann G, Stangl K, Stangl V (2009). Green and black tea are equally potent stimuli of NO production and vasodilation: new insights into tea ingredients involved. Basic Res Cardiol.

[19] Hajiaghaalipour F, Sanusi J, Kanthimathi MS (2016). Temperature and time of steeping affect the antioxidant Properties of White, Green, and Black Tea Infusions. J Food Sci.

[20] Li W, Yang J, Zhu XS, Li SC, Ho PC (2016). Correlation between tea consumption and prevalence of hypertension among singaporean chinese residents aged 40 years. J Hum Hypertens.

[21] Yang Y-C, Lu F-H, Wu J-S, Wu C-H, Chang C-J. (2004) The Protective Effect of Habitual Tea Consumption on Hypertension. Archives of internal medicine 164 (14):1534–1540. 10.1001/archinte.164.14.1534 Archives of Internal Medicine 10.1001/archinte.164.14.1534 15277285

[22] Liu G, Mi XN, Zheng XX, Xu YL, Lu J, Huang XH (2014). Effects of tea intake on blood pressure: a meta-analysis of randomised controlled trials. Br J Nutr.

[23] Grosso G, Stepaniak U, Micek A, Topor-Madry R, Pikhart H, Szafraniec K, Pajak A (2015). Association of daily coffee and tea consumption and metabolic syndrome: results from the polish arm of the HAPIEE study. Eur J Nutr.

[24] Gaeini Z, Bahadoran Z, Mirmiran P, Azizi F (2019). Tea, coffee, caffeine intake and the risk of cardio-metabolic outcomes: findings from a population with low coffee and high tea consumption. Nutr Metab (Lond).

[25] Bingham SA, Jerling HVJC, Magee E, Mulligan A, Runswick SA, Cummings JH (1997). Effect of black tea drinking on blood lipids, blood pressure and aspects of bowel habit. Br J Nutr.

[26] Feng C, Cao Y, Su Y, Cai H, Shu XO, Zheng W, Yu D, Zong G (2021). Association between Tea Consumption and Hypertension Risk among Middle-Aged and older chinese adults. J Nutr.

[27] Mahdavi-Roshan M, Salari A, Ghorbani Z, Ashouri A (2020). The effects of regular consumption of green or black tea beverage on blood pressure in those with elevated blood pressure or hypertension: a systematic review and meta-analysis. Complement Ther Med.

[28] Guan X, Yang J. Comparative study of tea consumption between urban and rural residents in China. Journal of Tea Science; 2015.

[29] Lu J, Lu Y, Wang X, Li X, Linderman GC, Wu C, Cheng X, Mu L, Zhang H, Liu J, Su M, Zhao H, Spatz ES, Spertus JA, Masoudi FA, Krumholz HM, Jiang L (2017). Prevalence, awareness, treatment, and control of hypertension in China: data from 1·7 million adults in a population-based screening study (China PEACE million persons project). The Lancet.

[30] Zhao X, Hong F, Yin J, Tang W, Zhang G, Liang X, Li J, Cui C, Li X (2020). Cohort profile: the China multi-ethnic cohort (CMEC) study. Int J Epidemiol.

[31] Perloff D, Grim C, Flack J, Frohlich ED, Hill M, McDonald M, Morgenstern BZ (1993). Human blood pressure determination by sphygmomanometry. Circulation.

[32] Yin J-Y, Liu S-YDF-C, Yao Q-K, Tu S, Xu Y, Pan C-W (2017). Blood pressure is Associated with Tea Consumption: a cross-sectional study in a Rural, Elderly Population of Jiangsu China. J Nutr Health Aging.

[33] Grassi D, Draijer R, Desideri G, Mulder T, Ferri C (2015). Black tea lowers blood pressure and wave reflections in fasted and postprandial conditions in hypertensive patients: a randomised study. Nutrients.

[34] Alkerwi A, Sauvageot N, Crichton GE, Elias MF (2015). Tea, but not coffee consumption, is associated with components of arterial pressure. The Observation of Cardiovascular Risk factors study in Luxembourg. Nutr Res.

[35] Niu XG, Cai C, Liu FC, Li JX, Huang KY, Yang XL, Cao J, Chen SF, Li HF, Shen C, Zhao YX, Hu DS, Gu SJ, Huang JF, Lu XF, Gu DF (2021). Associations of tea consumption with blood pressure progression and hypertension incidence. J geriatric cardiology: JGC.

[36] Pon Velayutham AB, Dongmin L (2008). Green Tea Catechins and Cardiovascular Health an Update. Curr Med Chem.

[37] Moore RJ, Jackson KG, Minihane AM (2009). Green tea (Camellia sinensis) catechins and vascular function. Br J Nutr.

[38] Gong J, Peng C, Chen T, Gao B, Zhou H (2010). Effects of theabrownin from pu-erh tea on the metabolism of serum lipids in rats: mechanism of action. J Food Sci.

[39] Li L, Shi M, Salerno S, Tang M, Guo F, Liu J, Feng Y, Fu M, Huang Q, Ma L, Li Y, Fu P (2019). Microbial and metabolomic remodeling by a formula of Sichuan dark tea improves hyperlipidemia in apoe-deficient mice. PLoS ONE.

[40] Lin F, Wei X-L, Liu H, Li H, Xia Y, Wu D, Zhang P, Gandhi G, Li H-B, Gan R-Y. State-of-the-art review of dark tea: from chemistry to health benefits. Trends Food Sci Technol. 2021;109. 10.1016/j.tifs.2021.01.030

[41] Yingzi H, Xiao W, Chuanshun L, Wen L, Qingsong P, Jinzhong Q. (2020) Study on the Antioxidant Activity of Wild Dark Tea. The wind of science and technology (33):157–159

[42] Cao SY, Li BY, Gan RY, Mao QQ, Wang YF, Shang A, Meng JM, Xu XY, Wei XL, Li HB. The in vivo antioxidant and hepatoprotective actions of selected chinese teas. Foods. 2020;9(3). 10.3390/foods9030262 10.3390/foods9030262 PMC7143450 32121649

[43] Luo D, Chen X, Zhu X, Liu S, Li J, Xu J, Zhao J, Ji X (2019). Pu-Erh Tea relaxes the thoracic aorta of rats by reducing intracellular calcium. Front Pharmacol.

[44] Ahmad AF, Rich L, Koch H, Croft KD, Ferruzzi MG, Kay CD, Hodgson JM, Ward NC (2018). Effect of adding milk to black tea on vascular function in healthy men and women: a randomised controlled crossover trial. Food Funct.

[45] Piovesana A, Rodrigues E, Norena CPZ (2019). Composition analysis of carotenoids and phenolic compounds and antioxidant activity from hibiscus calyces (Hibiscus sabdariffa L.) by HPLC-DAD-MS/MS. Phytochem Anal.

[46] Xu JW, Ikeda K, Yamori Y (2004). Upregulation of endothelial nitric oxide synthase by cyanidin-3-glucoside, a typical anthocyanin pigment. Hypertension.

[47] Ojeda D, Jimenez-Ferrer E, Zamilpa A, Herrera-Arellano A, Tortoriello J, Alvarez L (2010). Inhibition of angiotensin convertin enzyme (ACE) activity by the anthocyanins delphinidin- and cyanidin-3-O-sambubiosides from Hibiscus sabdariffa. J Ethnopharmacol.

[48] Jimenez-Ferrer E, Alarcon-Alonso J, Aguilar-Rojas A, Zamilpa A, Jimenez-Ferrer CI, Tortoriello J, Herrera-Ruiz M (2012). Diuretic effect of compounds from Hibiscus sabdariffa by modulation of the aldosterone activity. Planta Med.

[49] Liu RH (2003). Health benefits of fruit and vegetables are from additive and synergistic combinations of phytochemicals. Am J Clin Nutr.

[50] Xiaoliang Tong AWT, Lynne Giles GA, Wittert Z. Shi (2014 Oct 14) Tea consumption is inversely related to 5-year blood pressure change among adults in Jiangsu, China: a cross-sectional study. Nutr J. 10.1186/1475-2891-13-98 10.1186/1475-2891-13-98 PMC4209085 25311544

