# Supplementary material for: The association between tea consumption and blood pressure in the adult population in Southwest China
Source: BMC Public Health. 2023 Mar 13;23:476. doi: 10.1186/s12889-023-15315-5 (PMC10010002; doi:10.1186/s12889-023-15315-5)
Supplement: Supplementary file 1 — Supplementary Material 1 [file 12889_2023_15315_MOESM1_ESM.docx]

**The association between** **tea consumption and blood pressure in the adult population in Southwest China**

Ying Zhao^1,#^, Chengmeng Tang^1,#^, Wenge Tang^2,#^, Xuehui Zhang^3^, Xiaoman Jiang^4^, Zhuoma Duoji^5^, Yixi Kangzhu^6^, Xing Zhao^1^, Xiaohe Xu^7,8^, Feng Hong^9,*^, Qiaolan Liu^1,*^. The authors report no conflicts of interest.

^1^ West China School of Public Health and West China Fourth Hospital, Sichuan university, Chengdu 610041, China.

^2^ Chongqing Municipal Center for Disease Control and Prevention, Chongqing 400042, China.

^3^ School of Public Health, Kunming Medical University, Kunming 650500, China.

^4^ Chengdu Center for Disease Control and Prevention, Chengdu 610041, China.

^5^ School of Medicine, Tibet University, Lhasa 850000, China.

^6^ Tibet Center for disease control and prevention, Lhasa 850000, China.

^7^ Department of Sociology, University of Texas at San Antonio, San Antonio, USA.

^8^ Department of Sociology and Psychology, School of Public Administration, Sichuan University, 610064, Chengdu, China

^9^ School of Public Health, the Key Laboratory of Environmental Pollution Monitoring and Disease Control, Ministry of Education, Guizhou Medical University, Guiyang, 550025, China.

# Ying Zhao, Chengmeng Tang, and Wenge Tang have contributed equally to this work and share first authorship.

* Corresponding authors

Feng Hong, School of Public Health, the Key Laboratory of Environmental Pollution Monitoring and Disease Control, Ministry of Education, Guizhou Medical University, Guiyang 550025, China

mail addresses: fhong@gmc.edu.cn

Qiaolan Liu, West China School of Public Health and West China Fourth Hospital, Sichuan University, Chengdu City, Sichuan 610041, China

mail addresses: [liuqiaol@scu.edu.cn](mailto:liuqiaol@scu.edu.cn)

* These authors have contributed equally to this work and share corresponding authorship

The China multi-ethnic cohort (CMEC) was conducted from May 2018 to September 2019 in five provinces (Sichuan, Chongqing, Tibet, Guizhou, and Yunnan) in southwest China with a multistage stratified whole-group sampling. Han, Dong, Bouyi, Yi, Miao, Bai and Tibetan ethnic groups were included. Han Chinese are mainly distributed in Chengdu, Chongqing, and Lijiang, Yunnan; Tibetans are mainly distributed in Lhasa and Aba; Yi are mainly distributed in Chuxiong, Yunnan; Bai are distributed in Dali, Yunnan; Miao and Dong are distributed in Qiandongnan, Guizhou; and Bouyi are distributed in Qiannan, Guizhou.

Reference:

Zhao X, Hong F, Yin J, Tang W, Zhang G, Liang X, Li J, Cui C, Li X: Cohort profile: the China Multi-Ethnic cohort (CMEC) study. Int J Epidemiol 2020.

Table S1 Baseline characteristics of the participants with hypertensive BP

|  | **Hypertensive BP** | | **Total** | **P** |
| --- | --- | --- | --- | --- |
|  | **Yes (N1=14,624)** | **No(N2=62,049)** | **(N=76,673)** |  |
| **Age group (%)** |  |  |  | <0.001 |
| 30-39 | 1135(7.76) | 14574 (23.49) | 15709 (20.49) |  |
| 40-49 | 3817(26.10) | 22146 (35.69) | 25963 (33.86) |  |
| 50-59 | 4656(31.84) | 15161 (24.43) | 19817 (25.85) |  |
| 60-69 | 3636(24.86) | 7965 (12.84) | 11601 (15.13) |  |
| 70-79 | 1380(9.44) | 2203(3.55) | 3583(4.67) |  |
| **Sex (%)** |  |  |  | <0.001 |
| Male | 7,253(49.60) | 22,447(36.18) | 29,700(38.74) |  |
| Female | 7371(50.40) | 39602 (63.82) | 46973 (61.26) |  |
| **Ethnicity (%)** |  |  |  | <0.001 |
| Han | 8520(58.26) | 35953 (57.94) | 44473 (58.09) |  |
| Dong | 977(6.68) | 4363(7.03) | 5340(6.96) |  |
| Bouyi | 974(6.66) | 3612(5.82) | 4586(5.98) |  |
| Yi | 1091(7.46) | 3732(6.01) | 4823(6.29) |  |
| Miao | 866(5.92) | 3262(5.26) | 4128(5.38) |  |
| Bai | 994(6.80) | 3865(6.23) | 4859(6.34) |  |
| Tibetan | 1202(8.22) | 7262 (11.70) | 8464 (11.04) |  |
| **Marital status (%)** |  |  |  | <0.001 |
| Married | 12760(87.25) | 56034 (90.31) | 68794 (89.73) |  |
| Divorce | 575(3.93) | 2583(4.16) | 3158(4.12) |  |
| Widowed | 1096(7.49) | 2596(4.18) | 3692(4.82) |  |
| Single | 193(1.32) | 835(1.35) | 1028(1.34) |  |
| **Education (%)** |  |  |  | <0.001 |
| Illiteracy | 4164(28.47) | 15088 (24.32) | 19252 (25.11) |  |
| Primary | 3973(27.17) | 15026 (24.22) | 18999 (24.78) |  |
| Middle school | 5337(36.49) | 23900 (38.52) | 29237 (38.13) |  |
| College | 1150(7.86) | 8034 (12.95) | 9184 (11.98) |  |
| **Occupation (%)** |  |  |  | <0.001 |
| Employed | 12232(83.69) | 54809 (88.41) | 67041 (87.51) |  |
| Unemployed | 628(4.30) | 2806(4.53) | 3434(4.48) |  |
| Retired | 1756(12.01) | 4379(7.06) | 6135(8.01) |  |
| **Family income (RMB /year) (%)** |  |  |  | <0.001 |
| <12,000 | 2800(19.17) | 9583 (15.46) | 12383 (16.17) |  |
| 12,000-19,999 | 2772(18.98) | 11173 (18.03) | 13945 (18.21) |  |
| 20,000-59,999 | 5261(36.01) | 22845 (36.87) | 28106 (36.70) |  |
| 60,000-99,999 | 2037(13.94) | 9345 (15.08) | 11382 (14.86) |  |
| 100,000-199,999 | 1419(9.71) | 7151 (11.54) | 8570 (11.19) |  |
| ≥200,000 | 319(2.18) | 1872(3.02) | 2191(2.86) |  |
| **BMI (kg/m^2^) (%)** |  |  |  | <0.001 |
| Normal | 5742(39.34) | 34644 (55.92) | 40386 (52.76) |  |
| Overweight | 6171(42.28) | 21278 (34.34) | 27449 (35.86) |  |
| Obesity | 2682(18.38) | 6035(9.74) | 8717 (11.39) |  |
| **MET (%)** |  |  |  | <0.001 |
| Low | 4253(29.21) | 14765 (23.91) | 19018 (24.92) |  |
| Middle | 6975(47.90) | 31244 (50.59) | 38219 (50.08) |  |
| High | 3333(22.89) | 15750 (25.50) | 19083 (25.00) |  |
| **Smoking status (%)** |  |  |  | <0.001 |
| No | 10194(69.71) | 47808 (77.05) | 58002 (75.65) |  |
| Current | 3520(24.07) | 11784 (18.99) | 15304 (19.96) |  |
| Quit | 910(6.22) | 2457(3.96) | 3367(4.39) |  |
| **Alcohol use status (%)** |  |  |  | <0.001 |
| No | 7502(51.30) | 35770 (57.65) | 43272 (56.44) |  |
| Occasionally | 4147(28.36) | 19599 (31.59) | 23746 (30.97) |  |
| Frequently | 2975(20.34) | 6680 (10.77) | 9655 (12.59) |  |
| **Salt** **(g/w, mean±SD)** | 48.65±30.06 | 45.99±28.40 | 46.50±28.74 | <0.001 |
| **Vegetables (g/w, mean±SD)** | 2,236.82±1,485.66 | 2,144.84±1,441.01 | 2,162.39±1,450.08 | <0.001 |
| **Fruits (g/w, mean±SD)** | 753.42±770.94 | 867.12±812.62 | 845.42±806.07 | <0.001 |
| **Dairy (g/w, mean±SD)** | 335.28±566.79 | 401.22±590.21 | 388.64±586.38 | <0.001 |
| **Family history of hypertension (%)** |  |  |  | 0.019 |
| No | 11189(76.51) | 48035(77.41) | 59,224(77.24) |  |
| Yes | 3435(23.49) | 14014 (22.59) | 17449 (22.76) |  |
| **Physician-diagnosed disease (%)** |  |  |  | <0.001 |
| No | 11,759(80.41) | 51344(82.75) | 63103(82.30) |  |
| Yes | 2865(19.59) | 10705 (17.25) | 13570 (17.70) |  |
| **Status of tea consumption** |  |  |  | 0.017 |
| No | 9,673(66.14) | 41,685(67.18) | 51,358(66.98) |  |
| Yes | 4951(33.86) | 20364 (32.82) | 25315 (33.02) |  |
| **Types of tea consumption** |  |  |  | <0.001 |
| No | 9683(66.21) | 41714 (67.23) | 51397 (67.03) |  |
| Green tea | 2622(17.93) | 9374 (15.11) | 11996 (15.65) |  |
| Scented tea | 1018(6.96) | 3502(5.64) | 4520(5.90) |  |
| Dark tea | 723(4.94) | 4230(6.82) | 4953(6.46) |  |
| Sweet tea | 332(2.27) | 2109(3.40) | 2441(3.18) |  |
| Black tea | 112(0.77) | 598(0.96) | 710(0.93) |  |
| Other tea | 134(0.92) | 522(0.84) | 656(0.86) |  |
| **Duration of tea consumption (years) (%)** |  |  |  | <0.001 |
| No | 9675(66.16) | 41688 (67.19) | 51363 (66.99) |  |
| ≤15 | 1122(7.67) | 5042(8.13) | 6164(8.04) |  |
| 16-40 | 2531(17.31) | 11409 (18.39) | 13940 (18.18) |  |
| >40 | 1296(8.86) | 3910(6.30) | 5206(6.79) |  |
| **Frequency of tea consumption (d/w) (%)** |  |  |  | <0.001 |
| No | 9772(66.82) | 42149 (67.93) | 51921 (67.72) |  |
| 1-2 | 451(3.08) | 2180(3.51) | 2631(3.43) |  |
| 3-5 | 622(4.25) | 2738(4.41) | 3360(4.38) |  |
| 6-7 | 3779(25.84) | 14982 (24.15) | 18761 (24.47) |  |
| **Amount of tea consumption (g/d) (%)** |  |  |  | 0.014 |
| No | 9700(66.33) | 41748 (67.28) | 51448 (67.10) |  |
| ≤2.0 | 1253(8.57) | 5368(8.65) | 6621(8.64) |  |
| 2.1-4.0 | 1291(8.83) | 5477(8.83) | 6768(8.83) |  |
| 4.1-8.0 | 1210(8.27) | 4637(7.47) | 5847(7.63) |  |
| ≥8.1 | 1170(8.00) | 4819(7.77) | 5989(7.81) |  |

Table S2 Relationships between variables of tea consumption habits and SBP, DBP according to sex, BMI, smoking status, alcohol use status, age, and salt

| **Sex** | | | | | | | | | | | |
| --- | --- | --- | --- | --- | --- | --- | --- | --- | --- | --- | --- |
|  | Green tea |  | Scented tea |  | Dark tea |  | Sweet tea |  | Black tea |  | Other tea |
|  | β(95%CI) |  | β(95%CI) |  | β(95%CI) |  | β(95%CI) |  | β(95%CI) |  | β(95%CI) |
| **Male** | | | | | | | | | | | |
| **Outcome = SBP** | | | | | | | | | | | |
| **Duration of tea consumption (years)** | | | | | | | | | | | |
| ≤15 | -0.54(-1.27,0.19) |  | -1.63(-2.78,-0.48) |  | -0.75(-2.80,1.30) |  | -4.42(-9.02,0.17) |  | -0.45(-2.72,1.81) |  | 0.43(-1.88,2.73) |
| 16-40 | -1.45(-2.00,-0.91) |  | -1.44(-2.18,-0.69) |  | -1.74(-2.89,-0.60) |  | -4.01(-5.88,-2.13) |  | -1.41(-3.56,0.75) |  | -1.32(-3.82,1.18) |
| >40 | -1.89(-2.85,-0.94) |  | 0.14(-1.28,1.55) |  | -2.42(-3.86,-0.98) |  | -5.07(-7.02,-3.12) |  | -3.36(-7.95,1.23) |  | -3.65(-7.88,0.58) |
| **Frequency of tea consumption (d/w)** | | | | | | | | | | | |
| 1-2 | -1.73(-2.84,-0.62) |  | -2.51(-4.23,-0.78) |  | -2.08(-4.87,0.71) |  | -5.66(-11.49,0.18) |  | -2.12(-6.53,2.28) |  | -2.14(-6.55,2.27) |
| 3-5 | -0.71(-1.66,0.24) |  | -1.40(-2.74,-0.07) |  | 2.14(-0.33,4.62) |  | -5.27(-10.22,-0.33) |  | -1.01(-4.42,2.40) |  | -1.95(-5.50,1.61) |
| 6-7 | -1.29(-1.79,-0.79) |  | -1.07(-1.78,-0.36) |  | -2.08(-3.10,-1.06) |  | -4.37(-6.01,-2.73) |  | -1.01(-2.81,0.79) |  | -0.45(-2.41,1.51) |
| **Amount of tea consumption (g/d)** | | | | | | | | | | | |
| ≤2.0 | -1.94(-2.83,-1.05) |  | -1.98(-3.07,-0.89) |  | -4.35(-6.18,-2.52) |  | -3.83(-5.71,-1.95) |  | -2.14(-6.37,2.09) |  | -2.84(-6.19,0.52) |
| 2.1-4.0 | -1.30(-1.99,-0.61) |  | -1.14(-2.16,-0.13) |  | -3.56(-5.26,-1.85) |  | -5.55(-8.02,-3.07) |  | -2.25(-5.25,0.74) |  | -1.49(-4.58,1.61) |
| 4.1-8.0 | -1.23(-1.94,-0.53) |  | -1.64(-2.74,-0.54) |  | -0.30(-1.77,1.16) |  | -5.14(-8.38,-1.90) |  | -1.76(-4.64,1.12) |  | 0.09(-3.18,3.37) |
| ≥8.1 | -0.80(-1.53,-0.07) |  | -0.06(-1.25,1.13) |  | -0.89(-2.16,0.37) |  | -4.72(-7.24,-2.20) |  | 0.15(-2.27,2.57) |  | 0.25(-2.64,3.15) |
| **Outcome= DBP** | | | | | | | | | | | |
| **Duration of tea consumption (years)** | | | | | | | | | | | |
| ≤15 | 0.28(-0.21,0.76) |  | 0.42(-0.35,1.19) |  | 0.67(-0.70,2.05) |  | -0.63(-3.72,2.45) |  | 0.18(-1.34,1.70) |  | 0.19(-1.35,1.74) |
| 16-40 | 0.20(-0.15,0.56) |  | 0.27(-0.22,0.77) |  | -0.66(-1.43,0.11) |  | -0.95(-2.21,0.31) |  | -0.30(-1.75,1.14) |  | -0.42(-2.09,1.25) |
| >40 | 0.26(-0.37,0.89) |  | 0.68(-0.26,1.63) |  | 0.48(-0.49,1.45) |  | 0.75(-0.56,2.07) |  | 0.37(-2.70,3.45) |  | 0.14(-2.69,2.97) |
| **Frequency of tea consumption (**d/w**)** | | | | | | | | | | | |
| 1-2 | -0.36(-1.09,0.37) |  | -0.77(-1.92,0.38) |  | 0.09(-1.78,1.97) |  | -1.96(-5.88,1.96) |  | -1.74(-4.70,1.21) |  | -0.79(-3.74,2.16) |
| 3-5 | 0.47(-0.15,1.10) |  | 0.28(-0.62,1.17) |  | 0.86(-0.80,2.53) |  | -0.99(-4.31,2.33) |  | 0.67(-1.61,2.96) |  | -0.90(-3.28,1.47) |
| 6-7 | 0.31(-0.02,0.64) |  | 0.58(0.11,1.05) |  | -0.26(-0.94,0.43) |  | -0.06(-1.16,1.04) |  | 0.24(-0.96,1.45) |  | 0.20(-1.11,1.51) |
| **Amount of tea consumption (**g/d**)** | | | | | | | | | | | |
| ≤2.0 | -0.04(-0.63,0.55) |  | 0.08(-0.64,0.81) |  | 0.42(-0.81,1.65) |  | 0.77(-0.49,2.04) |  | 0.34(-2.49,3.18) |  | -0.17(-2.42,2.07) |
| 2.1-4.0 | -0.04(-0.50,0.41) |  | 0.27(-0.40,0.95) |  | -0.50(-1.65,0.64) |  | -0.68(-2.34,0.99) |  | -0.57(-2.58,1.44) |  | -0.52(-2.59,1.55) |
| 4.1-8.0 | 0.44(-0.02,0.91) |  | 0.23(-0.51,0.96) |  | -0.12(-1.11,0.87) |  | -0.80(-2.98,1.38) |  | 0.41(-1.52,2.34) |  | 0.29(-1.90,2.48) |
| ≥8.1 | 0.48(0.00,0.96) |  | 1.06(0.26,1.85) |  | -0.13(-0.98,0.72) |  | -1.72(-3.41,-0.03) |  | -0.02(-1.64,1.60) |  | 0.06(-1.88,1.99) |
|  | Green tea |  | Scented tea |  | Dark tea |  | Sweet tea |  | Black tea |  | Other tea |
|  | β(95%CI) |  | β(95%CI) |  | β(95%CI) |  | β(95%CI) |  | β(95%CI) |  | β(95%CI) |
| **Female** | | | | | | | | | | | |
| **Outcome = SBP** | | | | | | | | | | | |
| **Duration of tea consumption (years)** | | | | | | | | | | | |
| ≤15 | -0.50(-1.28,0.28) |  | -1.67(-2.85,-0.49) |  | -4.06(-5.79,-2.34) |  | -5.92(-9.08,-2.76) |  | -1.60(-4.02,0.81) |  | 2.17(-0.23,4.58) |
| 16-40 | -1.59(-2.24,-0.93) |  | -2.10(-3.31,-0.89) |  | -1.48(-2.50,-0.46) |  | -5.77(-7.16,-4.38) |  | -3.71(-6.55,-0.88) |  | -0.63(-3.55,2.29) |
| >40 | -1.79(-3.13,-0.44) |  | -2.91(-6.40,0.58) |  | -4.61(-5.82,-3.39) |  | -5.58(-7.08,-4.07) |  | -6.20(-10.92,-1.48) |  | -5.67(-10.37,-0.96) |
| **Frequency of tea consumption (d/w)** | | | | | | | | | | | |
| 1-2 | -1.10(-2.24,0.05) |  | -2.66(-4.54,-0.78) |  | -2.49(-4.65,-0.33) |  | -5.20(-9.48,-0.92) |  | 0.62(-2.97,4.21) |  | -0.14(-3.90,3.61) |
| 3-5 | -0.93(-2.08,0.21) |  | -1.05(-2.67,0.57) |  | -1.87(-3.94,0.20) |  | -6.67(-10.58,-2.76) |  | -4.14(-8.02,-0.26) |  | 1.56(-2.61,5.73) |
| 6-7 | -1.39(-1.98,-0.79) |  | -2.24(-3.38,-1.09) |  | -3.10(-4.02,-2.19) |  | -5.70(-6.90,-4.51) |  | -4.11(-6.40,-1.83) |  | -0.38(-2.68,1.92) |
| **Amount of tea consumption (g/d)** | | | | | | | | | | | |
| ≤2.0 | -1.49(-2.29,-0.69) |  | -1.83(-3.01,-0.65) |  | -5.72(-7.11,-4.34) |  | -6.11(-7.54,-4.69) |  | -2.44(-5.43,0.56) |  | -2.54(-5.32,0.24) |
| 2.1-4.0 | -1.53(-2.33,-0.73) |  | -1.58(-3.12,-0.04) |  | -4.85(-6.24,-3.46) |  | -8.05(-10.05,-6.04) |  | -3.45(-6.28,-0.63) |  | 1.54(-1.76,4.83) |
| 4.1-8.0 | -0.52(-1.53,0.50) |  | -2.69(-4.83,-0.55) |  | 0.18(-1.09,1.46) |  | -7.24(-9.74,-4.73) |  | -4.16(-8.69,0.38) |  | 3.68(-0.41,7.77) |
| ≥8.1 | -0.83(-2.24,0.57) |  | -2.12(-4.74,0.49) |  | -2.13(-3.29,-0.97) |  | -2.22(-4.09,-0.36) |  | -2.01(-6.31,2.29) |  | 0.22(-4.08,4.53) |
| **Outcome= DBP** | | | | | | | | | | | |
| **Duration of tea consumption (years)** | | | | | | | | | | | |
| ≤15 | 0.50(0.01,0.98) |  | 0.05(-0.69,0.79) |  | -1.82(-2.90,-0.74) |  | 2.37(0.39,4.35) |  | -1.22(-2.73,0.29) |  | 0.48(-1.02,1.98) |
| 16-40 | 0.70(0.29,1.11) |  | -0.49(-1.25,0.26) |  | -1.08(-1.72,-0.44) |  | -0.35(-1.22,0.52) |  | -0.83(-2.60,0.94) |  | 0.65(-1.17,2.47) |
| >40 | 1.26(0.42,2.10) |  | -2.12(-4.30,0.06) |  | 0.63(-0.13,1.40) |  | 2.17(1.22,3.11) |  | -1.09(-4.04,1.86) |  | 0.03(-2.90,2.97) |
| **Frequency of tea consumption (**d/w**)** | | | | | | | | | | | |
| 1-2 | 0.42(-0.30,1.13) |  | -0.94(-2.12,0.23) |  | -0.80(-2.15,0.55) |  | 0.69(-1.99,3.38) |  | -0.71(-2.95,1.53) |  | -0.73(-3.08,1.61) |
| 3-5 | 0.26(-0.46,0.97) |  | -0.19(-1.20,0.82) |  | -2.24(-3.53,-0.94) |  | -0.21(-2.66,2.24) |  | -1.28(-3.70,1.15) |  | 1.69(-0.92,4.29) |
| 6-7 | 0.90(0.53,1.27) |  | -0.05(-0.76,0.67) |  | -0.51(-1.09,0.06) |  | 0.89(0.14,1.65) |  | -1.23(-2.66,0.20) |  | 0.50(-0.93,1.94) |
| **Amount of tea consumption (**g/d**)** | | | | | | | | | | | |
| ≤2.0 | 0.63(0.13,1.13) |  | 0.23(-0.51,0.96) |  | 0.19(-0.68,1.06) |  | 1.68(0.79,2.58) |  | -1.71(-3.59,0.16) |  | 0.68(-1.05,2.42) |
| 2.1-4.0 | 0.39(-0.11,0.89) |  | -0.11(-1.07,0.85) |  | -1.25(-2.13,-0.38) |  | -0.86(-2.12,0.39) |  | -0.76(-2.53,1.00) |  | 1.05(-1.00,3.11) |
| 4.1-8.0 | 1.20(0.57,1.84) |  | -1.28(-2.62,0.06) |  | -0.25(-1.04,0.55) |  | -0.25(-1.82,1.32) |  | -1.03(-3.86,1.80) |  | 1.81(-0.75,4.36) |
| ≥8.1 | 0.74(-0.14,1.62) |  | -2.11(-3.75,-0.48) |  | -1.36(-2.09,-0.64) |  | 1.08(-0.09,2.25) |  | 0.07(-2.61,2.76) |  | -2.51(-5.20,0.17) |
| **BMI** | | | | | | | | | | | |
|  | Green tea |  | Scented tea |  | Dark tea |  | Sweet tea |  | Black tea |  | Other tea |
|  | β(95%CI) |  | β(95%CI) |  | β(95%CI) |  | β(95%CI) |  | β(95%CI) |  | β(95%CI) |
| **Normal** | | | | | | | | | | | |
| **Outcome = SBP** | | | | | | | | | | | |
| **Duration of tea consumption (years)** | | | | | | | | | | | |
| ≤15 | 0.03(-0.70,0.76) |  | -1.98(-3.13,-0.83) |  | -2.30(-4.21,-0.38) |  | -7.10(-11.89,-2.32) |  | -0.91(-3.17,1.36) |  | 0.19(-2.28,2.66) |
| 16-40 | -1.64(-2.20,-1.09) |  | -1.53(-2.45,-0.61) |  | -2.16(-3.26,-1.07) |  | -4.37(-6.34,-2.40) |  | -3.28(-5.72,-0.84) |  | -1.49(-4.69,1.72) |
| >40 | -2.51(-3.44,-1.57) |  | -1.86(-3.72,0.00) |  | -5.41(-6.86,-3.96) |  | -6.42(-8.58,-4.26) |  | -7.65(-12.67,-2.62) |  | -7.00(-13.00,-1.01) |
| **Frequency of tea consumption (d/w)** | | | | | | | | | | | |
| 1-2 | -1.04(-2.13,0.05) |  | -1.73(-3.58,0.12) |  | -2.41(-4.84,0.03) |  | -5.48(-12.64,1.68) |  | -2.03(-5.84,1.79) |  | -3.35(-7.67,0.97) |
| 3-5 | -0.95(-1.98,0.07) |  | -0.89(-2.41,0.63) |  | -1.13(-3.46,1.21) |  | -5.03(-12.63,2.58) |  | -3.14(-6.54,0.25) |  | -0.44(-4.46,3.59) |
| 6-7 | -1.48(-1.98,-0.99) |  | -1.96(-2.82,-1.11) |  | -3.41(-4.40,-2.42) |  | -5.42(-7.07,-3.78) |  | -2.54(-4.59,-0.50) |  | -0.64(-3.12,1.84) |
| **Amount of tea consumption (g/d)** | | | | | | | | | | | |
| ≤2.0 | -1.33(-2.13,-0.54) |  | -2.25(-3.38,-1.12) |  | -5.20(-6.95,-3.45) |  | -5.34(-7.56,-3.12) |  | -1.78(-4.91,1.35) |  | -4.87(-8.40,-1.34) |
| 2.1-4.0 | -1.22(-1.91,-0.53) |  | -1.54(-2.77,-0.31) |  | -4.61(-6.18,-3.05) |  | -8.45(-11.15,-5.74) |  | -3.02(-6.03,0.00) |  | 0.09(-3.67,3.84) |
| 4.1-8.0 | -1.16(-1.92,-0.39) |  | -2.46(-3.91,-1.01) |  | -1.53(-2.96,-0.10) |  | -5.70(-9.10,-2.30) |  | -3.64(-7.07,-0.20) |  | 0.58(-3.00,4.16) |
| ≥8.1 | -1.53(-2.40,-0.66) |  | -0.06(-1.66,1.53) |  | -1.95(-3.23,-0.66) |  | -2.68(-5.23,-0.13) |  | -1.90(-4.96,1.17) |  | 0.53(-3.54,4.59) |
| **Outcome= DBP** | | | | | | | | | | | |
| **Duration of tea consumption (years)** | | | | | | | | | | | |
| ≤15 | 0.70(0.23,1.16) |  | 0.16(-0.58,0.89) |  | -0.11(-1.34,1.11) |  | 0.49(-2.56,3.55) |  | -0.22(-1.67,1.22) |  | -0.40(-1.98,1.17) |
| 16-40 | 0.40(0.04,0.75) |  | -0.12(-0.71,0.47) |  | -0.94(-1.64,-0.24) |  | -0.29(-1.54,0.96) |  | -0.62(-2.18,0.93) |  | -0.29(-2.33,1.75) |
| >40 | 0.72(0.13,1.32) |  | -0.07(-1.26,1.12) |  | 0.06(-0.87,0.99) |  | 1.24(-0.14,2.61) |  | -1.58(-4.78,1.63) |  | -1.11(-4.93,2.71) |
| **Frequency of tea consumption (**d/w**)** | | | | | | | | | | | |
| 1-2 | 0.35(-0.34,1.04) |  | -0.59(-1.77,0.59) |  | -0.05(-1.60,1.51) |  | -2.37(-6.94,2.19) |  | -1.83(-4.27,0.60) |  | -2.34(-5.09,0.41) |
| 3-5 | 0.23(-0.42,0.88) |  | -0.22(-1.19,0.75) |  | -0.83(-2.32,0.67) |  | 2.76(-2.10,7.61) |  | -0.05(-2.21,2.11) |  | -0.65(-3.22,1.91) |
| 6-7 | 0.65(0.34,0.97) |  | 0.32(-0.22,0.87) |  | -0.57(-1.21,0.06) |  | 0.42(-0.64,1.47) |  | -0.32(-1.62,0.99) |  | 0.33(-1.25,1.91) |
| **Amount of tea consumption (**g/d**)** | | | | | | | | | | | |
| ≤2.0 | 0.58(0.08,1.09) |  | -0.36(-1.09,0.36) |  | 0.08(-1.04,1.20) |  | 1.91(0.49,3.33) |  | -0.79(-2.78,1.21) |  | -0.48(-2.73,1.77) |
| 2.1-4.0 | 0.34(-0.10,0.78) |  | 0.29(-0.49,1.07) |  | -0.87(-1.87,0.14) |  | -1.94(-3.66,-0.21) |  | -0.55(-2.47,1.37) |  | 1.34(-1.05,3.73) |
| 4.1-8.0 | 0.80(0.31,1.28) |  | -0.62(-1.54,0.30) |  | -0.78(-1.69,0.14) |  | 0.47(-1.70,2.64) |  | 0.06(-2.13,2.25) |  | -0.30(-2.58,1.98) |
| ≥8.1 | 0.53(-0.02,1.09) |  | 0.91(-0.11,1.92) |  | -0.48(-1.30,0.34) |  | 0.20(-1.43,1.83) |  | -0.64(-2.60,1.31) |  | -2.61(-5.21,-0.02) |
|  | Green tea |  | Scented tea |  | Dark tea |  | Sweet tea |  | Black tea |  | Other tea |
|  | β(95%CI) |  | β(95%CI) |  | β(95%CI) |  | β(95%CI) |  | β(95%CI) |  | β(95%CI) |
| **Overweight** | | | | | | | | | | | |
| **Outcome = SBP** | | | | | | | | | | | |
| **Duration of tea consumption (years)** | | | | | | | | | | | |
| ≤15 | -0.58(-1.48,0.31) |  | -0.79(-2.16,0.59) |  | -2.98(-5.09,-0.88) |  | -4.00(-7.55,-0.45) |  | -0.65(-3.36,2.06) |  | 3.41(0.80,6.02) |
| 16-40 | -2.24(-2.93,-1.56) |  | -2.06(-3.04,-1.08) |  | -1.52(-2.84,-0.21) |  | -4.46(-6.12,-2.81) |  | -1.17(-4.01,1.68) |  | -0.75(-3.60,2.09) |
| >40 | -2.65(-4.12,-1.18) |  | -1.90(-3.95,0.15) |  | -2.41(-3.93,-0.88) |  | -4.50(-6.23,-2.76) |  | -2.25(-7.34,2.84) |  | -5.00(-9.14,-0.85) |
| **Frequency of tea consumption (d/w)** | | | | | | | | | | | |
| 1-2 | -1.89(-3.21,-0.57) |  | -3.41(-5.40,-1.42) |  | -2.85(-5.66,-0.04) |  | -3.09(-7.64,1.46) |  | 0.01(-4.61,4.64) |  | 0.74(-3.75,5.22) |
| 3-5 | -0.35(-1.55,0.84) |  | -0.92(-2.56,0.71) |  | -0.58(-3.34,2.19) |  | -5.07(-9.02,-1.12) |  | -0.24(-4.77,4.29) |  | 1.31(-3.17,5.79) |
| 6-7 | -2.11(-2.75,-1.46) |  | -1.64(-2.59,-0.68) |  | -2.07(-3.23,-0.92) |  | -4.43(-5.91,-2.95) |  | -1.48(-3.74,0.78) |  | -0.41(-2.58,1.75) |
| **Amount of tea consumption (g/d)** | | | | | | | | | | | |
| ≤2.0 | -2.21(-3.23,-1.20) |  | -1.77(-3.04,-0.49) |  | -4.92(-6.61,-3.24) |  | -4.57(-6.20,-2.94) |  | -3.83(-8.03,0.38) |  | -0.19(-3.32,2.93) |
| 2.1-4.0 | -2.28(-3.17,-1.40) |  | -1.30(-2.62,0.03) |  | -3.78(-5.60,-1.97) |  | -5.42(-7.64,-3.19) |  | -2.57(-5.81,0.68) |  | -0.89(-4.25,2.48) |
| 4.1-8.0 | -1.38(-2.37,-0.39) |  | -2.85(-4.42,-1.29) |  | 0.84(-0.78,2.47) |  | -4.64(-7.54,-1.74) |  | -2.01(-5.99,1.96) |  | 3.85(-0.32,8.02) |
| ≥8.1 | -1.05(-2.12,0.02) |  | -0.49(-2.25,1.27) |  | -1.59(-3.03,-0.14) |  | -3.08(-5.29,-0.86) |  | 2.65(-0.81,6.11) |  | -0.35(-3.91,3.20) |
| **Outcome= DBP** | | | | | | | | | | | |
| **Duration of tea consumption (years)** | | | | | | | | | | | |
| ≤15 | 0.40(-0.17,0.98) |  | 0.46(-0.42,1.33) |  | -1.38(-2.73,-0.04) |  | 1.74(-0.54,4.02) |  | -0.62(-2.35,1.11) |  | 2.04(0.38,3.70) |
| 16-40 | 0.16(-0.27,0.60) |  | 0.31(-0.32,0.93) |  | -0.99(-1.83,-0.16) |  | -0.25(-1.32,0.81) |  | 0.46(-1.36,2.28) |  | 0.29(-1.52,2.10) |
| >40 | -0.11(-1.05,0.82) |  | 0.49(-0.81,1.80) |  | 1.26(0.28,2.23) |  | 2.03(0.92,3.14) |  | 0.55(-2.70,3.80) |  | -0.26(-2.90,2.38) |
| **Frequency of tea consumption (**d/w**)** | | | | | | | | | | | |
| 1-2 | -0.31(-1.15,0.54) |  | -1.03(-2.30,0.23) |  | -0.51(-2.30,1.29) |  | 1.08(-1.84,4.00) |  | -1.31(-4.27,1.64) |  | -0.35(-3.21,2.50) |
| 3-5 | 0.67(-0.09,1.44) |  | 0.80(-0.24,1.84) |  | -1.89(-3.66,-0.12) |  | -0.88(-3.42,1.66) |  | 0.30(-2.59,3.19) |  | 2.41(-0.44,5.27) |
| 6-7 | 0.22(-0.19,0.63) |  | 0.47(-0.14,1.07) |  | -0.25(-0.99,0.49) |  | 0.90(-0.05,1.85) |  | 0.42(-1.02,1.86) |  | 0.76(-0.62,2.14) |
| **Amount of tea consumption (**g/d**)** | | | | | | | | | | | |
| ≤2.0 | 0.20(-0.45,0.85) |  | 0.70(-0.11,1.51) |  | 0.59(-0.49,1.66) |  | 1.29(0.25,2.34) |  | -1.63(-4.32,1.05) |  | 1.59(-0.40,3.58) |
| 2.1-4.0 | -0.30(-0.86,0.27) |  | 0.03(-0.81,0.88) |  | -0.79(-1.95,0.37) |  | 0.27(-1.16,1.70) |  | -0.20(-2.28,1.87) |  | -0.83(-2.97,1.32) |
| 4.1-8.0 | 0.54(-0.09,1.17) |  | 0.12(-0.88,1.12) |  | -0.05(-1.09,0.99) |  | 0.79(-1.08,2.65) |  | -0.33(-2.87,2.21) |  | 2.82(0.16,5.47) |
| ≥8.1 | 0.42(-0.27,1.10) |  | 0.78(-0.34,1.90) |  | -1.06(-1.99,-0.14) |  | 0.05(-1.37,1.48) |  | 1.69(-0.51,3.90) |  | 0.77(-1.49,3.04) |
|  | Green tea |  | Scented tea |  | Dark tea |  | Sweet tea |  | Black tea |  | Other tea |
|  | β(95%CI) |  | β(95%CI) |  | β(95%CI) |  | β(95%CI) |  | β(95%CI) |  | β(95%CI) |
| **Obesity** | | | | | | | | | | | |
| **Outcome = SBP** | | | | | | | | | | | |
| **Duration of tea consumption (years)** | | | | | | | | | | | |
| ≤15 | -0.61(-2.20,0.98) |  | -1.54(-3.90,0.82) |  | -2.08(-5.89,1.73) |  | -3.66(-10.69,3.37) |  | -0.56(-6.28,5.15) |  | 0.79(-4.01,5.59) |
| 16-40 | -1.53(-2.91,-0.16) |  | -2.80(-4.64,-0.96) |  | -0.70(-2.58,1.18) |  | -5.07(-7.87,-2.27) |  | -4.10(-8.98,0.78) |  | -1.85(-6.40,2.70) |
| >40 | -6.27(-9.35,-3.18) |  | -4.90(-8.89,-0.92) |  | -3.70(-5.83,-1.57) |  | -4.62(-7.67,-1.57) |  | -9.06(-18.08,-0.04) |  | 0.77(-8.61,10.15) |
| **Frequency of tea consumption (d/w)** | | | | | | | | | | | |
| 1-2 | -1.06(-3.64,1.52) |  | -3.55(-7.48,0.38) |  | -0.63(-5.43,4.17) |  | -8.04(-16.70,0.61) |  | 3.12(-5.81,12.05) |  | 3.05(-4.69,10.79) |
| 3-5 | -0.91(-3.09,1.26) |  | -3.89(-6.80,-0.98) |  | 1.73(-1.88,5.34) |  | -6.11(-13.15,0.94) |  | -4.06(-12.39,4.27) |  | -2.09(-8.87,4.68) |
| 6-7 | -1.60(-2.87,-0.34) |  | -2.06(-3.79,-0.32) |  | -2.25(-3.94,-0.55) |  | -4.62(-6.99,-2.25) |  | -4.78(-8.98,-0.58) |  | -0.47(-4.51,3.56) |
| **Amount of tea consumption (g/d)** | | | | | | | | | | | |
| ≤2.0 | -3.21(-5.18,-1.23) |  | -4.07(-6.66,-1.49) |  | -4.57(-7.54,-1.59) |  | -3.28(-6.27,-0.29) |  | -5.73(-16.69,5.23) |  | -4.05(-9.93,1.82) |
| 2.1-4.0 | -1.71(-3.45,0.04) |  | -3.13(-5.70,-0.56) |  | -5.21(-7.94,-2.49) |  | -8.35(-12.55,-4.15) |  | -4.83(-10.84,1.19) |  | 1.13(-4.45,6.72) |
| 4.1-8.0 | -1.72(-3.64,0.20) |  | 0.38(-2.27,3.04) |  | 0.70(-1.56,2.95) |  | -11.61(-16.45,-6.77) |  | -1.44(-8.96,6.09) |  | -0.70(-8.99,7.59) |
| ≥8.1 | 0.13(-1.86,2.13) |  | -3.54(-6.39,-0.69) |  | -1.24(-3.23,0.74) |  | -1.30(-5.05,2.44) |  | -2.49(-8.30,3.32) |  | 1.58(-4.41,7.57) |
| **Outcome= DBP** | | | | | | | | | | | |
| **Duration of tea consumption (years)** | | | | | | | | | | | |
| ≤15 | -0.05(-1.07,0.96) |  | 0.45(-1.05,1.96) |  | -0.96(-3.41,1.48) |  | 3.49(-1.00,7.97) |  | 0.24(-3.38,3.86) |  | -1.23(-4.27,1.80) |
| 16-40 | 0.40(-0.48,1.28) |  | -0.21(-1.39,0.96) |  | -0.89(-2.10,0.32) |  | -0.59(-2.37,1.19) |  | -3.01(-6.10,0.08) |  | 0.00(-2.87,2.88) |
| >40 | -1.58(-3.54,0.39) |  | -2.59(-5.13,-0.05) |  | 0.04(-1.33,1.41) |  | 2.66(0.72,4.61) |  | -0.84(-6.55,4.87) |  | 5.77(-0.17,11.70) |
| **Frequency of tea consumption (**d/w**)** | | | | | | | | | | | |
| 1-2 | -0.06(-1.70,1.58) |  | -1.42(-3.92,1.08) |  | -1.67(-4.75,1.41) |  | 0.11(-5.42,5.63) |  | 2.23(-3.42,7.88) |  | 3.52(-1.37,8.42) |
| 3-5 | 1.01(-0.37,2.39) |  | -0.71(-2.56,1.14) |  | -0.10(-2.41,2.22) |  | 0.35(-4.15,4.84) |  | -2.33(-7.60,2.94) |  | -1.22(-5.50,3.07) |
| 6-7 | -0.04(-0.84,0.76) |  | 0.14(-0.96,1.25) |  | -0.48(-1.57,0.60) |  | 1.06(-0.45,2.57) |  | -2.28(-4.94,0.38) |  | -0.53(-3.08,2.03) |
| **Amount of tea consumption (**g/d**)** | | | | | | | | | | | |
| ≤2.0 | -0.72(-1.98,0.53) |  | -0.41(-2.05,1.24) |  | 0.38(-1.53,2.30) |  | 3.38(1.48,5.29) |  | -2.34(-9.27,4.60) |  | -0.68(-4.40,3.04) |
| 2.1-4.0 | 0.11(-1.00,1.22) |  | -0.27(-1.90,1.37) |  | -1.68(-3.43,0.08) |  | -0.98(-3.66,1.70) |  | -3.05(-6.85,0.76) |  | 1.17(-2.36,4.70) |
| 4.1-8.0 | 0.16(-1.07,1.38) |  | 0.81(-0.88,2.50) |  | 0.41(-1.04,1.86) |  | -4.36(-7.44,-1.27) |  | 1.29(-3.47,6.05) |  | 1.13(-4.11,6.38) |
| ≥8.1 | 0.53(-0.74,1.80) |  | -1.25(-3.07,0.56) |  | -1.08(-2.36,0.20) |  | 1.18(-1.20,3.57) |  | -1.10(-4.78,2.57) |  | -0.82(-4.61,2.97) |
| **Smoking status** | | | | | | | | | | | |
|  | Green tea |  | Scented tea |  | Dark tea |  | Sweet tea |  | Black tea |  | Other tea |
|  | β(95%CI) |  | β(95%CI) |  | β(95%CI) |  | β(95%CI) |  | β(95%CI) |  | β(95%CI) |
| **No** | | | | | | | | | | | |
| **Outcome = SBP** | | | | | | | | | | | |
| **Duration of tea consumption (years)** | | | | | | | | | | | |
| ≤15 | -0.43(-1.09,0.23) |  | -1.30(-2.35,-0.25) |  | -3.46(-5.00,-1.93) |  | -4.86(-7.81,-1.92) |  | -1.25(-3.29,0.80) |  | 2.32(0.28,4.36) |
| 16-40 | -1.77(-2.31,-1.23) |  | -2.38(-3.32,-1.43) |  | -1.33(-2.20,-0.45) |  | -5.38(-6.62,-4.13) |  | -2.71(-5.01,-0.41) |  | -0.53(-2.90,1.84) |
| >40 | -2.56(-3.66,-1.47) |  | -2.74(-5.04,-0.45) |  | -4.14(-5.18,-3.11) |  | -5.59(-6.91,-4.26) |  | -5.25(-9.26,-1.23) |  | -5.14(-8.95,-1.33) |
| **Frequency of tea consumption (d/w)** | | | | | | | | | | | |
| 1-2 | -1.39(-2.37,-0.41) |  | -2.88(-4.48,-1.27) |  | -2.52(-4.46,-0.58) |  | -4.93(-8.85,-1.01) |  | 0.04(-3.19,3.28) |  | -0.38(-3.64,2.88) |
| 3-5 | -0.81(-1.73,0.11) |  | -1.00(-2.38,0.37) |  | -0.98(-2.75,0.78) |  | -6.30(-9.75,-2.86) |  | -3.26(-6.34,-0.18) |  | 0.21(-3.05,3.47) |
| 6-7 | -1.61(-2.10,-1.11) |  | -2.14(-3.05,-1.23) |  | -2.76(-3.55,-1.98) |  | -5.43(-6.49,-4.37) |  | -2.73(-4.62,-0.85) |  | 0.21(-1.68,2.11) |
| **Amount of tea consumption (g/d)** | | | | | | | | | | | |
| ≤2.0 | -1.68(-2.40,-0.97) |  | -2.27(-3.31,-1.23) |  | -5.72(-6.96,-4.49) |  | -5.80(-7.08,-4.53) |  | -2.58(-5.37,0.20) |  | -2.79(-5.27,-0.31) |
| 2.1-4.0 | -1.66(-2.33,-0.99) |  | -2.04(-3.27,-0.81) |  | -4.50(-5.72,-3.29) |  | -7.46(-9.25,-5.67) |  | -3.21(-5.65,-0.78) |  | 1.28(-1.45,4.00) |
| 4.1-8.0 | -0.85(-1.64,-0.05) |  | -2.02(-3.63,-0.42) |  | 0.21(-0.86,1.28) |  | -6.62(-8.92,-4.31) |  | -1.60(-4.96,1.76) |  | 2.82(-0.29,5.94) |
| ≥8.1 | -1.12(-2.14,-0.10) |  | -0.58(-2.47,1.32) |  | -1.76(-2.74,-0.77) |  | -2.56(-4.21,-0.91) |  | -1.05(-4.17,2.06) |  | 0.85(-2.56,4.26) |
| **Outcome= DBP** | | | | | | | | | | | |
| **Duration of tea consumption (years)** | | | | | | | | | | | |
| ≤15 | 0.47(0.05,0.89) |  | 0.17(-0.49,0.83) |  | -1.32(-2.29,-0.34) |  | 2.47(0.60,4.34) |  | -0.70(-2.00,0.59) |  | 1.05(-0.24,2.34) |
| 16-40 | 0.49(0.14,0.83) |  | -0.56(-1.16,0.04) |  | -1.07(-1.63,-0.51) |  | -0.46(-1.25,0.34) |  | -0.34(-1.80,1.11) |  | 0.84(-0.66,2.34) |
| >40 | 0.57(-0.12,1.26) |  | -0.59(-2.04,0.87) |  | 0.49(-0.17,1.15) |  | 1.80(0.96,2.64) |  | 0.34(-2.20,2.88) |  | 0.11(-2.30,2.52) |
| **Frequency of tea consumption (**d/w**)** | | | | | | | | | | | |
| 1-2 | 0.21(-0.41,0.83) |  | -1.15(-2.16,-0.13) |  | -0.96(-2.19,0.27) |  | 0.84(-1.65,3.33) |  | -1.09(-3.14,0.95) |  | -0.46(-2.52,1.60) |
| 3-5 | 0.40(-0.18,0.98) |  | 0.01(-0.86,0.88) |  | -1.73(-2.85,-0.61) |  | 0.09(-2.09,2.28) |  | -0.83(-2.78,1.12) |  | 0.21(-1.85,2.27) |
| 6-7 | 0.62(0.30,0.93) |  | -0.03(-0.61,0.55) |  | -0.51(-1.01,-0.02) |  | 0.70(0.02,1.37) |  | -0.06(-1.25,1.13) |  | 1.44(0.24,2.64) |
| **Amount of tea consumption (g/d)** | | | | | | | | | | | |
| ≤2.0 | 0.53(0.08,0.98) |  | -0.02(-0.68,0.64) |  | 0.24(-0.54,1.03) |  | 1.61(0.80,2.42) |  | -1.67(-3.43,0.09) |  | 0.61(-0.96,2.18) |
| 2.1-4.0 | 0.18(-0.25,0.60) |  | -0.46(-1.24,0.32) |  | -1.29(-2.06,-0.51) |  | -0.91(-2.04,0.23) |  | -0.85(-2.40,0.69) |  | 1.51(-0.21,3.23) |
| 4.1-8.0 | 0.89(0.39,1.40) |  | -0.63(-1.65,0.39) |  | -0.32(-1.00,0.36) |  | -0.09(-1.55,1.37) |  | 1.39(-0.74,3.52) |  | 1.90(-0.07,3.87) |
| ≥8.1 | 0.47(-0.18,1.12) |  | 0.00(-1.20,1.20) |  | -1.04(-1.67,-0.42) |  | 0.47(-0.58,1.51) |  | 0.64(-1.33,2.61) |  | -1.16(-3.31,1.00) |
|  | Green tea |  | Scented tea |  | Dark tea |  | Sweet tea |  | Black tea |  | Other tea |
|  | β(95%CI) |  | β(95%CI) |  | β(95%CI) |  | β(95%CI) |  | β(95%CI) |  | β(95%CI) |
| **Current** | | | | | | | | | | | |
| **Outcome = SBP** | | | | | | | | | | | |
| **Duration of tea consumption (years)** | | | | | | | | | | | |
| ≤15 | -0.94(-1.93,0.05) |  | -2.10(-3.56,-0.65) |  | -0.45(-3.41,2.51) |  | -6.26(-12.33,-0.19) |  | -0.03(-3.12,3.05) |  | -1.26(-4.42,1.89) |
| 16-40 | -1.50(-2.22,-0.77) |  | -1.05(-2.02,-0.08) |  | -2.84(-4.61,-1.06) |  | -3.80(-7.11,-0.48) |  | -3.16(-6.00,-0.32) |  | -1.47(-5.10,2.16) |
| >40 | -1.38(-2.66,-0.11) |  | 0.57(-1.40,2.53) |  | -2.43(-5.00,0.14) |  | -3.97(-7.54,-0.39) |  | -5.09(-12.10,1.92) |  | -3.95(-11.02,3.11) |
| **Frequency of tea consumption (d/w)** | | | | | | | | | | | |
| 1-2 | -1.49(-3.01,0.03) |  | -2.72(-5.07,-0.37) |  | -1.18(-5.49,3.13) |  | -3.44(-11.47,4.59) |  | -1.96(-7.91,3.99) |  | -2.59(-8.91,3.73) |
| 3-5 | -1.00(-2.35,0.35) |  | -0.89(-2.61,0.84) |  | 3.56(-0.73,7.86) |  | -4.60(-12.07,2.87) |  | 0.05(-5.23,5.33) |  | -0.69(-5.95,4.57) |
| 6-7 | -1.38(-2.04,-0.72) |  | -1.09(-2.00,-0.18) |  | -2.86(-4.42,-1.30) |  | -3.89(-7.00,-0.77) |  | -2.38(-4.73,-0.02) |  | -1.86(-4.64,0.92) |
| **Amount of tea consumption (g/d)** | | | | | | | | | | | |
| ≤2.0 | -2.49(-3.71,-1.26) |  | -1.34(-2.79,0.10) |  | -1.85(-4.69,0.99) |  | -3.04(-6.38,0.30) |  | -1.32(-7.15,4.51) |  | -2.90(-8.04,2.24) |
| 2.1-4.0 | -1.43(-2.37,-0.50) |  | -0.65(-1.97,0.68) |  | -3.30(-6.10,-0.49) |  | -4.95(-8.98,-0.91) |  | -3.49(-7.77,0.78) |  | -2.95(-7.55,1.64) |
| 4.1-8.0 | -1.33(-2.26,-0.40) |  | -1.94(-3.32,-0.55) |  | -1.42(-3.99,1.16) |  | -4.83(-9.54,-0.12) |  | -4.28(-8.23,-0.32) |  | -1.18(-6.07,3.70) |
| ≥8.1 | -0.76(-1.67,0.16) |  | -0.49(-1.96,0.99) |  | -2.36(-4.38,-0.33) |  | -5.70(-10.07,-1.33) |  | 0.02(-3.08,3.13) |  | -0.28(-4.05,3.48) |
| **Outcome= DBP** | | | | | | | | | | | |
| **Duration of tea consumption (years)** | | | | | | | | | | | |
| ≤15 | 0.07(-0.58,0.72) |  | 0.15(-0.81,1.11) |  | 1.23(-0.74,3.21) |  | -2.93(-6.98,1.13) |  | 0.32(-1.73,2.37) |  | -1.20(-3.29,0.89) |
| 16-40 | 0.08(-0.40,0.55) |  | 0.39(-0.25,1.03) |  | -0.41(-1.60,0.77) |  | -1.37(-3.58,0.85) |  | -1.44(-3.32,0.45) |  | -1.20(-3.61,1.21) |
| >40 | 0.62(-0.21,1.45) |  | 1.05(-0.25,2.35) |  | 0.68(-1.03,2.40) |  | 0.55(-1.84,2.94) |  | -3.56(-8.22,1.10) |  | 0.92(-3.76,5.61) |
| **Frequency of tea consumption (**d/w**)** | | | | | | | | | | | |
| 1-2 | -0.59(-1.59,0.40) |  | -0.80(-2.35,0.75) |  | 1.68(-1.19,4.56) |  | -3.36(-8.73,2.01) |  | -1.61(-5.56,2.34) |  | -1.90(-6.09,2.29) |
| 3-5 | 0.23(-0.66,1.11) |  | 0.55(-0.59,1.69) |  | 3.05(0.19,5.91) |  | -3.28(-8.28,1.71) |  | 1.60(-1.91,5.11) |  | 0.87(-2.62,4.36) |
| 6-7 | 0.29(-0.14,0.73) |  | 0.53(-0.07,1.13) |  | -0.25(-1.29,0.79) |  | -0.47(-2.55,1.61) |  | -1.14(-2.70,0.43) |  | -1.49(-3.34,0.35) |
| **Amount of tea consumption (**g/d**)** | | | | | | | | | | | |
| ≤2.0 | -0.69(-1.49,0.12) |  | 0.25(-0.70,1.21) |  | 1.30(-0.59,3.19) |  | 0.10(-2.13,2.34) |  | 0.95(-2.92,4.83) |  | -1.37(-4.78,2.04) |
| 2.1-4.0 | -0.12(-0.73,0.49) |  | 0.64(-0.24,1.51) |  | 0.60(-1.27,2.46) |  | -1.17(-3.87,1.53) |  | -1.31(-4.15,1.53) |  | -2.48(-5.52,0.57) |
| 4.1-8.0 | 0.36(-0.25,0.97) |  | 0.09(-0.82,1.00) |  | 0.29(-1.42,2.01) |  | -2.41(-5.56,0.74) |  | -1.66(-4.29,0.97) |  | -0.47(-3.71,2.77) |
| ≥8.1 | 0.59(-0.01,1.18) |  | 0.71(-0.27,1.68) |  | -0.56(-1.90,0.79) |  | -2.20(-5.13,0.72) |  | -0.65(-2.71,1.41) |  | -0.10(-2.60,2.39) |
|  | Green tea |  | Scented tea |  | Dark tea |  | Sweet tea |  | Black tea |  | Other tea |
|  | β(95%CI) |  | β(95%CI) |  | β(95%CI) |  | β(95%CI) |  | β(95%CI) |  | β(95%CI) |
| **Quit** | | | | | | | | | | | |
| **Outcome = SBP** | | | | | | | | | | | |
| **Duration of tea consumption (years)** | | | | | | | | | | | |
| ≤15 | 2.32(0.04,4.60) |  | -1.43(-5.08,2.21) |  | 0.91(-4.81,6.63) |  | -11.70(-33.01,9.62) |  | -3.88(-11.59,3.83) |  | 5.80(-2.22,13.83) |
| 16-40 | -0.18(-1.90,1.53) |  | -1.21(-3.25,0.83) |  | -0.33(-3.71,3.06) |  | -0.53(-7.21,6.16) |  | 3.88(-2.99,10.75) |  | -2.88(-10.01,4.24) |
| >40 | -2.36(-4.76,0.05) |  | -0.22(-3.18,2.75) |  | -3.98(-7.95,0.00) |  | -5.81(-11.60,-0.01) |  | -3.78(-14.60,7.04) |  | -3.76(-13.38,5.86) |
| **Frequency of tea consumption (d/w)** | | | | | | | | | | | |
| 1-2 | -0.90(-4.18,2.37) |  | 1.21(-3.62,6.05) |  | -2.15(-9.09,4.80) |  | -22.19(-43.46,-0.91) |  | -3.56(-18.50,11.39) |  | -2.17(-23.21,18.86) |
| 3-5 | 0.66(-2.21,3.53) |  | -4.99(-8.99,-0.99) |  | 1.29(-5.78,8.35) |  | -9.02(-30.44,12.41) |  | 0.02(-10.50,10.55) |  | -6.09(-20.99,8.81) |
| 6-7 | 0.17(-1.35,1.70) |  | -0.61(-2.49,1.28) |  | -0.90(-3.93,2.13) |  | -3.51(-8.81,1.79) |  | -0.06(-5.82,5.70) |  | 0.62(-4.59,5.84) |
| **Amount of tea consumption (g/d)** | | | | | | | | | | | |
| ≤2.0 | 1.06(-1.36,3.47) |  | -1.80(-4.48,0.89) |  | -4.42(-9.71,0.88) |  | -1.64(-7.96,4.67) |  | -4.47(-15.76,6.82) |  | 0.97(-7.09,9.02) |
| 2.1-4.0 | 0.38(-1.66,2.43) |  | -0.81(-3.49,1.87) |  | -3.88(-8.44,0.69) |  | -6.40(-13.70,0.89) |  | 0.80(-8.59,10.19) |  | 1.76(-7.28,10.79) |
| 4.1-8.0 | -0.93(-3.17,1.31) |  | -0.68(-3.80,2.43) |  | -2.05(-7.01,2.91) |  | -7.65(-17.65,2.34) |  | -1.96(-10.57,6.66) |  | -3.28(-16.67,10.12) |
| ≥8.1 | -0.54(-2.96,1.88) |  | -0.42(-3.71,2.87) |  | 1.75(-1.86,5.37) |  | -4.03(-11.81,3.75) |  | 0.60(-8.08,9.27) |  | -1.97(-10.69,6.75) |
| **Outcome= DBP** | | | | | | | | | | | |
| **Duration of tea consumption (years)** | | | | | | | | | | | |
| ≤15 | 1.91(0.46,3.35) |  | 1.26(-1.10,3.63) |  | -0.69(-4.40,3.03) |  | 0.58(-13.19,14.36) |  | -3.04(-8.01,1.94) |  | 1.67(-3.48,6.83) |
| 16-40 | 1.52(0.43,2.61) |  | 0.98(-0.34,2.31) |  | 0.29(-1.91,2.49) |  | -0.25(-4.57,4.07) |  | 1.83(-2.61,6.26) |  | -1.38(-5.96,3.19) |
| >40 | 0.75(-0.77,2.28) |  | 0.25(-1.67,2.18) |  | 0.15(-2.43,2.74) |  | 0.65(-3.09,4.40) |  | 3.71(-3.27,10.69) |  | 0.11(-6.07,6.29) |
| **Frequency of tea consumption (**d/w**)** | | | | | | | | | | | |
| 1-2 | 1.36(-0.72,3.44) |  | 1.15(-1.99,4.28) |  | 0.67(-3.84,5.18) |  | -9.99(-23.73,3.75) |  | -0.25(-9.89,9.40) |  | 2.40(-11.09,15.90) |
| 3-5 | 1.85(0.03,3.67) |  | -1.70(-4.29,0.90) |  | -0.15(-4.74,4.43) |  | 0.16(-13.67,14.00) |  | 1.10(-5.69,7.89) |  | -0.28(-9.84,9.28) |
| 6-7 | 1.33(0.36,2.30) |  | 1.35(0.13,2.57) |  | 0.56(-1.41,2.52) |  | 0.56(-2.87,3.99) |  | 0.96(-2.76,4.68) |  | -0.20(-3.54,3.15) |
| **Amount of tea consumption (**g/d**)** | | | | | | | | | | | |
| ≤2.0 | 2.50(0.97,4.03) |  | 0.76(-0.98,2.51) |  | -1.53(-4.97,1.92) |  | 1.34(-2.73,5.42) |  | 1.84(-5.45,9.12) |  | 2.83(-2.34,7.99) |
| 2.1-4.0 | 1.39(0.09,2.68) |  | 0.89(-0.85,2.62) |  | -0.08(-3.05,2.89) |  | 0.43(-4.28,5.14) |  | 2.65(-3.41,8.70) |  | 0.64(-5.15,6.43) |
| 4.1-8.0 | 1.15(-0.27,2.57) |  | 1.12(-0.90,3.14) |  | 0.15(-3.07,3.37) |  | 1.56(-4.90,8.01) |  | -2.30(-7.86,3.25) |  | -4.49(-13.08,4.10) |
| ≥8.1 | 0.74(-0.79,2.28) |  | 0.62(-1.51,2.76) |  | 0.63(-1.72,2.98) |  | -2.35(-7.37,2.68) |  | 0.17(-5.43,5.76) |  | -2.02(-7.61,3.57) |
| **Alcohol use status** | | | | | | | | | | | |
|  | Green tea |  | Scented tea |  | Dark tea |  | Sweet tea |  | Black tea |  | Other tea |
|  | β(95%CI) |  | β(95%CI) |  | β(95%CI) |  | β(95%CI) |  | β(95%CI) |  | β(95%CI) |
| **No** | | | | | | | | | | | |
| **Outcome = SBP** | | | | | | | | | | | |
| **Duration of tea consumption (years)** | | | | | | | | | | | |
| ≤15 | -0.94(-1.77,-0.11) |  | -1.45(-2.79,-0.11) |  | -3.17(-4.99,-1.34) |  | -5.02(-8.33,-1.71) |  | -0.55(-3.58,2.48) |  | 2.08(-0.80,4.96) |
| 16-40 | -2.27(-2.88,-1.65) |  | -1.37(-2.44,-0.30) |  | -1.31(-2.22,-0.40) |  | -4.86(-6.19,-3.53) |  | -3.61(-6.35,-0.88) |  | -1.45(-4.49,1.59) |
| >40 | -2.75(-3.87,-1.62) |  | -2.27(-4.34,-0.19) |  | -3.98(-5.05,-2.90) |  | -6.19(-7.58,-4.81) |  | -7.69(-12.16,-3.21) |  | -4.76(-8.99,-0.53) |
| **Frequency of tea consumption (d/w)** | | | | | | | | | | | |
| 1-2 | -2.53(-3.73,-1.33) |  | -2.11(-4.12,-0.11) |  | -1.75(-3.86,0.37) |  | -3.95(-8.23,0.33) |  | -1.66(-6.39,3.06) |  | -2.36(-6.83,2.10) |
| 3-5 | -1.73(-2.97,-0.48) |  | -0.20(-2.03,1.63) |  | -0.29(-2.22,1.64) |  | -5.16(-9.18,-1.14) |  | -4.26(-8.99,0.47) |  | -1.22(-6.57,4.12) |
| 6-7 | -1.88(-2.43,-1.33) |  | -1.82(-2.82,-0.83) |  | -2.65(-3.47,-1.82) |  | -5.50(-6.61,-4.39) |  | -3.19(-5.44,-0.93) |  | -0.30(-2.65,2.04) |
| **Amount of tea consumption (g/d)** | | | | | | | | | | | |
| ≤2.0 | -1.90(-2.76,-1.03) |  | -1.73(-2.99,-0.46) |  | -6.03(-7.38,-4.68) |  | -5.75(-7.10,-4.40) |  | -3.53(-7.34,0.29) |  | -3.14(-6.15,-0.13) |
| 2.1-4.0 | -2.30(-3.07,-1.53) |  | -1.23(-2.67,0.21) |  | -4.63(-5.92,-3.34) |  | -7.21(-9.16,-5.26) |  | -3.89(-7.00,-0.77) |  | 1.56(-2.09,5.20) |
| 4.1-8.0 | -1.63(-2.53,-0.74) |  | -2.06(-3.87,-0.25) |  | 0.12(-1.01,1.24) |  | -7.16(-9.61,-4.71) |  | -4.74(-9.30,-0.18) |  | 1.33(-3.04,5.70) |
| ≥8.1 | -1.77(-2.84,-0.70) |  | -0.86(-2.72,1.00) |  | -1.32(-2.34,-0.30) |  | -2.88(-4.62,-1.13) |  | -0.78(-4.52,2.96) |  | -0.15(-4.72,4.41) |
| **Outcome= DBP** | | | | | | | | | | | |
| **Duration of tea consumption (years)** | | | | | | | | | | | |
| ≤15 | 0.24(-0.28,0.76) |  | 0.08(-0.76,0.91) |  | -1.84(-2.98,-0.70) |  | 1.65(-0.42,3.72) |  | -0.18(-2.07,1.70) |  | 0.72(-1.07,2.51) |
| 16-40 | 0.23(-0.15,0.62) |  | 0.08(-0.59,0.75) |  | -1.16(-1.73,-0.59) |  | -0.50(-1.34,0.33) |  | -0.59(-2.29,1.11) |  | 0.45(-1.45,2.34) |
| >40 | 0.53(-0.17,1.23) |  | -0.42(-1.71,0.87) |  | 0.46(-0.22,1.13) |  | 1.56(0.69,2.43) |  | -1.32(-4.10,1.47) |  | 0.27(-2.36,2.90) |
| **Frequency of tea consumption (**d/w**)** | | | | | | | | | | | |
| 1-2 | -0.41(-1.16,0.34) |  | -0.59(-1.84,0.66) |  | -1.01(-2.34,0.32) |  | 0.22(-2.46,2.89) |  | -1.46(-4.40,1.48) |  | -1.35(-4.13,1.42) |
| 3-5 | -0.02(-0.79,0.76) |  | 0.59(-0.55,1.73) |  | -1.86(-3.07,-0.66) |  | 0.21(-2.30,2.72) |  | -1.82(-4.76,1.12) |  | 1.09(-2.24,4.41) |
| 6-7 | 0.50(0.16,0.85) |  | 0.06(-0.56,0.68) |  | -0.59(-1.10,-0.07) |  | 0.55(-0.14,1.25) |  | -0.06(-1.46,1.35) |  | 0.87(-0.59,2.32) |
| **Amount of tea consumption (**g/d**)** | | | | | | | | | | | |
| ≤2.0 | 0.64(0.10,1.18) |  | -0.04(-0.83,0.74) |  | -0.35(-1.20,0.50) |  | 1.39(0.55,2.24) |  | -2.15(-4.52,0.22) |  | 0.44(-1.43,2.32) |
| 2.1-4.0 | 0.05(-0.43,0.53) |  | 0.37(-0.52,1.27) |  | -1.38(-2.19,-0.57) |  | -0.55(-1.77,0.67) |  | -0.03(-1.97,1.91) |  | 1.70(-0.57,3.97) |
| 4.1-8.0 | 0.45(-0.11,1.01) |  | -0.47(-1.60,0.66) |  | -0.29(-1.00,0.41) |  | -0.17(-1.70,1.36) |  | -2.15(-4.98,0.69) |  | 0.66(-2.05,3.38) |
| ≥8.1 | -0.09(-0.76,0.57) |  | 0.14(-1.02,1.30) |  | -0.93(-1.57,-0.29) |  | -0.06(-1.16,1.03) |  | 1.67(-0.65,4.00) |  | -1.26(-4.10,1.58) |
|  | Green tea |  | Scented tea |  | Dark tea |  | Sweet tea |  | Black tea |  | Other tea |
|  | β(95%CI) |  | β(95%CI) |  | β(95%CI) |  | β(95%CI) |  | β(95%CI) |  | β(95%CI) |
| **Yes** | | | | | | | | | | | |
| **Outcome = SBP** | | | | | | | | | | | |
| **Duration of tea consumption (years)** | | | | | | | | | | | |
| ≤15 | 0.35(-0.34,1.05) |  | -1.20(-2.23,-0.16) |  | -1.82(-3.74,0.11) |  | -3.83(-8.34,0.68) |  | -0.50(-2.46,1.45) |  | 1.27(-0.76,3.29) |
| 16-40 | -0.97(-1.53,-0.41) |  | -1.56(-2.33,-0.79) |  | -2.50(-3.95,-1.06) |  | -3.09(-5.69,-0.50) |  | -1.46(-3.66,0.73) |  | -0.31(-2.74,2.12) |
| >40 | -2.24(-3.29,-1.20) |  | -0.84(-2.49,0.82) |  | -4.22(-6.18,-2.26) |  | -2.04(-4.81,0.73) |  | -1.77(-6.69,3.15) |  | -4.71(-9.54,0.12) |
| **Frequency of tea consumption (d/w)** | | | | | | | | | | | |
| 1-2 | -0.30(-1.36,0.77) |  | -2.44(-4.09,-0.80) |  | -3.54(-6.47,-0.60) |  | -5.60(-11.75,0.55) |  | 0.35(-3.06,3.76) |  | 0.57(-3.14,4.27) |
| 3-5 | 0.04(-0.86,0.94) |  | -1.45(-2.70,-0.21) |  | -0.16(-3.04,2.72) |  | -6.04(-11.09,-0.98) |  | -0.90(-3.93,2.12) |  | 0.23(-2.88,3.33) |
| 6-7 | -1.00(-1.52,-0.48) |  | -1.15(-1.88,-0.41) |  | -2.95(-4.21,-1.68) |  | -2.56(-5.02,-0.11) |  | -1.41(-3.22,0.39) |  | -0.16(-2.10,1.77) |
| **Amount of tea consumption (g/d)** | | | | | | | | | | | |
| ≤2.0 | -1.50(-2.33,-0.68) |  | -2.04(-3.07,-1.01) |  | -2.70(-4.71,-0.70) |  | -1.89(-4.54,0.76) |  | -1.55(-4.72,1.61) |  | -1.66(-4.74,1.41) |
| 2.1-4.0 | -0.64(-1.35,0.06) |  | -1.29(-2.32,-0.25) |  | -3.78(-5.81,-1.74) |  | -4.69(-7.75,-1.63) |  | -1.73(-4.46,1.01) |  | -0.56(-3.42,2.30) |
| 4.1-8.0 | -0.57(-1.32,0.18) |  | -1.59(-2.74,-0.43) |  | -1.05(-3.05,0.94) |  | -2.84(-6.62,0.94) |  | -1.82(-4.68,1.04) |  | 2.00(-1.14,5.14) |
| ≥8.1 | -0.21(-1.01,0.59) |  | -0.03(-1.35,1.29) |  | -2.96(-4.60,-1.33) |  | -2.46(-5.76,0.85) |  | 0.60(-1.94,3.15) |  | 0.53(-2.28,3.33) |
| **Outcome= DBP** | | | | | | | | | | | |
| **Duration of tea consumption (years)** | | | | | | | | | | | |
| ≤15 | 0.76(0.30,1.21) |  | 0.68(0.00,1.36) |  | 0.46(-0.81,1.73) |  | 0.68(-2.30,3.66) |  | -0.30(-1.59,0.99) |  | 0.26(-1.08,1.60) |
| 16-40 | 0.62(0.26,0.99) |  | 0.54(0.04,1.05) |  | -0.24(-1.19,0.72) |  | -0.79(-2.51,0.93) |  | -0.32(-1.77,1.13) |  | 0.17(-1.43,1.77) |
| >40 | 0.66(-0.03,1.34) |  | 0.91(-0.19,2.00) |  | 0.45(-0.84,1.75) |  | 1.67(-0.16,3.51) |  | 0.97(-2.28,4.22) |  | -0.25(-3.43,2.93) |
| **Frequency of tea consumption (**d/w**)** | | | | | | | | | | | |
| 1-2 | 0.47(-0.23,1.17) |  | -0.74(-1.82,0.34) |  | 0.51(-1.43,2.45) |  | -1.18(-5.25,2.89) |  | -0.97(-3.22,1.28) |  | -0.18(-2.62,2.26) |
| 3-5 | 0.84(0.26,1.43) |  | 0.22(-0.60,1.04) |  | 1.06(-0.84,2.97) |  | -2.01(-5.35,1.34) |  | 0.73(-1.27,2.73) |  | 0.00(-2.04,2.05) |
| 6-7 | 0.69(0.35,1.03) |  | 1.07(0.59,1.56) |  | -0.12(-0.96,0.71) |  | 0.41(-1.21,2.04) |  | -0.22(-1.41,0.98) |  | 0.22(-1.05,1.50) |
| **Amount of tea consumption (**g/d**)** | | | | | | | | | | | |
| ≤2.0 | 0.12(-0.42,0.66) |  | 0.54(-0.14,1.22) |  | 1.73(0.40,3.05) |  | 1.29(-0.46,3.05) |  | -0.24(-2.33,1.85) |  | 0.30(-1.73,2.32) |
| 2.1-4.0 | 0.36(-0.11,0.82) |  | 0.39(-0.30,1.07) |  | 0.00(-1.34,1.34) |  | -1.19(-3.21,0.84) |  | -1.14(-2.95,0.66) |  | -0.36(-2.25,1.53) |
| 4.1-8.0 | 1.03(0.54,1.52) |  | 0.59(-0.17,1.35) |  | 0.09(-1.22,1.41) |  | -0.80(-3.30,1.71) |  | 0.91(-0.98,2.80) |  | 1.30(-0.77,3.38) |
| ≥8.1 | 1.14(0.62,1.67) |  | 1.26(0.39,2.13) |  | -0.66(-1.74,0.42) |  | 0.04(-2.15,2.22) |  | -0.17(-1.85,1.51) |  | -0.40(-2.25,1.45) |
| **Age** | | | | | | | | | | | |
|  | Green tea |  | Scented tea |  | Dark tea |  | Sweet tea |  | Black tea |  | Other tea |
|  | β(95%CI) |  | β(95%CI) |  | β(95%CI) |  | β(95%CI) |  | β(95%CI) |  | β(95%CI) |
| **< 50 years old** | | | | | | | | | | | |
| **Outcome = SBP** | | | | | | | | | | | |
| **Duration of tea consumption (years)** | | | | | | | | | | | |
| ≤15 | -0.18(-0.76,0.41) |  | -1.65(-2.55,-0.76) |  | -2.74(-4.17,-1.31) |  | -6.52(-9.18,-3.86) |  | -0.55(-2.20,1.11) |  | 0.47(-1.36,2.29) |
| 16-40 | -1.31(-1.81,-0.81) |  | -0.99(-1.78,-0.20) |  | -1.67(-2.51,-0.84) |  | -5.50(-6.62,-4.39) |  | -1.36(-3.19,0.47) |  | -0.77(-3.04,1.49) |
| >40 | -1.35(-3.94,1.23) |  | -3.80(-10.80,3.20) |  | -3.09(-4.86,-1.31) |  | -4.42(-6.23,-2.62) |  | -2.84(-9.43,3.75) |  | 0.42(-7.93,8.77) |
| **Frequency of tea consumption (d/w)** | | | | | | | | | | | |
| 1-2 | -0.84(-1.74,0.06) |  | -2.49(-3.90,-1.08) |  | -2.49(-4.40,-0.58) |  | -4.48(-8.10,-0.86) |  | -0.95(-3.82,1.93) |  | 0.68(-5.02,6.38) |
| 3-5 | -0.11(-0.92,0.70) |  | -0.70(-1.86,0.46) |  | 0.17(-1.55,1.88) |  | -7.25(-10.47,-4.03) |  | -0.71(-3.18,1.76) |  | 1.92(-3.30,7.13) |
| 6-7 | -1.08(-1.56,-0.60) |  | -1.02(-1.81,-0.22) |  | -2.38(-3.18,-1.57) |  | -5.35(-6.41,-4.28) |  | -0.94(-2.55,0.67) |  | -0.45(-2.86,1.97) |
| **Amount of tea consumption (g/d)** | | | | | | | | | | | |
| ≤2.0 | -1.07(-1.81,-0.33) |  | -1.33(-2.38,-0.28) |  | -5.71(-7.04,-4.38) |  | -5.55(-6.82,-4.29) |  | -1.17(-3.81,1.47) |  | -0.59(-3.41,2.23) |
| 2.1-4.0 | -1.16(-1.81,-0.51) |  | -1.16(-2.24,-0.09) |  | -4.62(-5.91,-3.32) |  | -8.38(-10.12,-6.65) |  | -2.76(-4.99,-0.52) |  | -1.43(-4.31,1.44) |
| 4.1-8.0 | -0.37(-1.09,0.35) |  | -1.57(-2.79,-0.36) |  | 0.50(-0.61,1.60) |  | -5.53(-7.68,-3.37) |  | -1.29(-4.02,1.45) |  | 3.28(0.31,6.24) |
| ≥8.1 | -0.69(-1.50,0.11) |  | -1.13(-2.46,0.19) |  | -0.76(-1.76,0.23) |  | -2.47(-4.12,-0.83) |  | 1.04(-1.14,3.23) |  | -0.86(-3.43,1.70) |
| **Outcome= DBP** | | | | | | | | | | | |
| **Duration of tea consumption (years)** | | | | | | | | | | | |
| ≤15 | 0.55(0.15,0.96) |  | 0.40(-0.23,1.02) |  | -0.95(-1.95,0.04) |  | 1.07(-0.78,2.93) |  | 0.17(-0.98,1.32) |  | 0.50(-0.77,1.77) |
| 16-40 | 0.66(0.31,1.01) |  | 0.99(0.44,1.54) |  | -0.49(-1.07,0.10) |  | -0.18(-0.96,0.59) |  | -0.19(-1.46,1.08) |  | 0.60(-0.98,2.17) |
| >40 | 0.64(-1.17,2.44) |  | -2.67(-7.55,2.21) |  | 0.22(-1.02,1.46) |  | 1.48(0.22,2.75) |  | 1.60(-2.99,6.19) |  | 3.95(-1.86,9.76) |
| **Frequency of tea consumption (**d/w**)** | | | | | | | | | | | |
| 1-2 | 0.39(-0.24,1.01) |  | -0.32(-1.31,0.66) |  | -0.26(-1.59,1.08) |  | 1.23(-1.29,3.76) |  | -0.96(-2.96,1.04) |  | -0.04(-3.34,3.27) |
| 3-5 | 0.79(0.22,1.36) |  | 0.71(-0.10,1.52) |  | -0.75(-1.95,0.44) |  | -1.08(-3.33,1.17) |  | 0.44(-1.28,2.16) |  | 2.04(-0.98,5.06) |
| 6-7 | 0.71(0.37,1.04) |  | 1.12(0.57,1.67) |  | -0.59(-1.15,-0.02) |  | 0.25(-0.50,0.99) |  | 0.42(-0.70,1.53) |  | -0.47(-1.87,0.93) |
| **Amount of tea consumption (**g/d**)** | | | | | | | | | | | |
| ≤2.0 | 0.62(0.11,1.14) |  | 0.90(0.16,1.63) |  | -0.01(-0.94,0.92) |  | 1.02(0.14,1.91) |  | -0.53(-2.36,1.31) |  | 1.38(-0.58,3.34) |
| 2.1-4.0 | 0.37(-0.09,0.82) |  | 0.90(0.15,1.65) |  | -1.32(-2.22,-0.41) |  | -1.73(-2.94,-0.52) |  | -0.96(-2.52,0.59) |  | 0.93(-1.08,2.93) |
| 4.1-8.0 | 0.91(0.41,1.42) |  | 0.35(-0.49,1.20) |  | -0.04(-0.82,0.73) |  | 0.35(-1.16,1.85) |  | 0.85(-1.05,2.75) |  | 0.61(-1.45,2.67) |
| ≥8.1 | 0.60(0.03,1.16) |  | 0.54(-0.38,1.47) |  | -0.73(-1.42,-0.04) |  | 0.04(-1.12,1.19) |  | 1.04(-0.48,2.56) |  | -0.20(-1.98,1.59) |
|  | Green tea |  | Scented tea |  | Dark tea |  | Sweet tea |  | Black tea |  | Other tea |
|  | β(95%CI) |  | β(95%CI) |  | β(95%CI) |  | β(95%CI) |  | β(95%CI) |  | β(95%CI) |
| **≥ 50 years old** | | | | | | | | | | | |
| **Outcome = SBP** | | | | | | | | | | | |
| **Duration of tea consumption (years)** | | | | | | | | | | | |
| ≤15 | -0.87(-1.92,0.17) |  | -2.26(-3.92,-0.60) |  | -3.82(-6.50,-1.13) |  | -5.70(-11.86,0.47) |  | -1.09(-5.34,3.16) |  | 3.66(0.34,6.97) |
| 16-40 | -2.30(-2.98,-1.62) |  | -2.19(-3.20,-1.17) |  | -3.44(-5.07,-1.81) |  | -7.70(-11.34,-4.06) |  | -4.35(-7.96,-0.73) |  | -0.52(-3.84,2.79) |
| >40 | -0.89(-1.80,0.03) |  | 0.55(-0.98,2.07) |  | -2.25(-3.65,-0.84) |  | -3.94(-5.88,-2.01) |  | -4.23(-8.49,0.03) |  | -3.98(-7.89,-0.06) |
| **Frequency of tea consumption (d/w)** | | | | | | | | | | | |
| 1-2 | -2.37(-3.85,-0.88) |  | -3.25(-5.73,-0.78) |  | -1.86(-5.10,1.38) |  | -7.93(-15.48,-0.37) |  | 2.82(-3.52,9.17) |  | 0.68(-5.02,6.38) |
| 3-5 | -1.30(-2.68,0.08) |  | -2.47(-4.41,-0.53) |  | -1.57(-4.82,1.68) |  | -2.50(-9.21,4.20) |  | -8.97(-16.81,-1.14) |  | 1.92(-3.30,7.13) |
| 6-7 | -1.61(-2.21,-1.02) |  | -1.40(-2.32,-0.48) |  | -2.95(-4.15,-1.75) |  | -4.42(-6.30,-2.54) |  | -3.90(-6.53,-1.26) |  | -0.45(-2.86,1.97) |
| **Amount of tea consumption (g/d)** | | | | | | | | | | | |
| ≤2.0 | -2.04(-3.01,-1.06) |  | -2.13(-3.38,-0.87) |  | -4.27(-6.17,-2.38) |  | -3.85(-6.03,-1.66) |  | -3.78(-8.77,1.21) |  | -3.59(-6.95,-0.22) |
| 2.1-4.0 | -1.50(-2.34,-0.65) |  | -1.41(-2.77,-0.05) |  | -4.24(-6.09,-2.39) |  | -4.99(-7.97,-2.02) |  | -2.51(-6.67,1.65) |  | 1.95(-1.70,5.61) |
| 4.1-8.0 | -1.74(-2.68,-0.80) |  | -2.08(-3.70,-0.46) |  | -1.08(-2.82,0.65) |  | -7.51(-11.49,-3.54) |  | -4.51(-9.17,0.15) |  | 0.25(-4.41,4.91) |
| ≥8.1 | -1.37(-2.39,-0.34) |  | 0.11(-1.70,1.93) |  | -2.59(-4.11,-1.06) |  | -4.03(-6.96,-1.10) |  | -3.01(-7.85,1.83) |  | 4.17(-0.94,9.27) |
| **Outcome= DBP** | | | | | | | | | | | |
| **Duration of tea consumption (years)** | | | | | | | | | | | |
| ≤15 | 0.37(-0.24,0.97) |  | -0.28(-1.24,0.69) |  | -0.64(-2.20,0.92) |  | 2.01(-1.58,5.59) |  | -1.98(-4.44,0.49) |  | 0.14(-1.78,2.06) |
| 16-40 | 0.65(0.25,1.04) |  | -0.02(-0.61,0.56) |  | -0.75(-1.69,0.20) |  | -0.73(-2.84,1.39) |  | 0.05(-2.05,2.15) |  | 0.48(-1.45,2.40) |
| >40 | 0.20(-0.33,0.73) |  | -0.13(-1.01,0.76) |  | -0.15(-0.97,0.67) |  | 1.26(0.14,2.39) |  | -1.30(-3.77,1.17) |  | -0.77(-3.04,1.50) |
| **Frequency of tea consumption (**d/w**)** | | | | | | | | | | | |
| 1-2 | -0.32(-1.18,0.54) |  | -2.02(-3.46,-0.59) |  | -0.28(-2.16,1.61) |  | -3.14(-7.54,1.25) |  | -1.40(-5.08,2.28) |  | -0.04(-3.34,3.27) |
| 3-5 | 0.36(-0.44,1.17) |  | -0.47(-1.60,0.65) |  | -1.22(-3.11,0.67) |  | 1.35(-2.55,5.24) |  | -2.43(-6.97,2.12) |  | 2.04(-0.98,5.06) |
| 6-7 | 0.57(0.23,0.92) |  | 0.22(-0.32,0.75) |  | -0.30(-1.00,0.40) |  | 1.11(0.02,2.21) |  | -0.80(-2.32,0.73) |  | -0.47(-1.87,0.93) |
| **Amount of tea consumption (**g/d**)** | | | | | | | | | | | |
| ≤2.0 | 0.29(-0.27,0.86) |  | -0.36(-1.09,0.37) |  | 0.51(-0.59,1.61) |  | 1.97(0.70,3.24) |  | -1.66(-4.55,1.23) |  | -0.58(-2.53,1.37) |
| 2.1-4.0 | 0.12(-0.38,0.61) |  | -0.24(-1.03,0.55) |  | -0.59(-1.67,0.48) |  | 0.91(-0.82,2.63) |  | 0.29(-2.12,2.71) |  | -0.11(-2.23,2.00) |
| 4.1-8.0 | 0.73(0.18,1.27) |  | -0.16(-1.10,0.79) |  | -0.30(-1.31,0.71) |  | -1.34(-3.65,0.97) |  | -0.84(-3.55,1.86) |  | 2.18(-0.52,4.88) |
| ≥8.1 | 0.84(0.24,1.43) |  | 0.95(-0.11,2.00) |  | -0.92(-1.81,-0.04) |  | 0.13(-1.57,1.83) |  | -1.69(-4.49,1.12) |  | -1.21(-4.17,1.75) |
| **Salt intake** | | | | | | | | | | | |
|  | Green tea |  | Scented tea |  | Dark tea |  | Sweet tea |  | Black tea |  | Other tea |
|  | β(95%CI) |  | β(95%CI) |  | β(95%CI) |  | β(95%CI) |  | β(95%CI) |  | β(95%CI) |
| **< 46.5 g/w** | | | | | | | | | | | |
| **Outcome = SBP** | | | | | | | | | | | |
| **Duration of tea consumption (years)** | | | | | | | | | | | |
| ≤15 | -0.21(-0.88,0.46) |  | -1.10(-2.09,-0.10) |  | -2.42(-3.97,-0.86) |  | -5.26(-8.54,-1.99) |  | -0.49(-2.45,1.47) |  | 2.00(-0.02,4.02) |
| 16-40 | -1.79(-2.31,-1.28) |  | -1.50(-2.27,-0.73) |  | -1.75(-2.66,-0.84) |  | -4.75(-6.07,-3.43) |  | -2.34(-4.40,-0.28) |  | -1.21(-3.55,1.13) |
| >40 | -2.74(-3.73,-1.75) |  | -2.50(-4.10,-0.89) |  | -4.17(-5.29,-3.06) |  | -6.00(-7.45,-4.55) |  | -6.78(-11.21,-2.34) |  | -3.83(-7.52,-0.13) |
| **Frequency of tea consumption (d/w)** | | | | | | | | | | | |
| 1-2 | -1.07(-2.08,-0.05) |  | -1.95(-3.46,-0.45) |  | -1.89(-3.90,0.12) |  | -5.85(-10.19,-1.51) |  | -0.84(-4.35,2.67) |  | -0.27(-3.70,3.15) |
| 3-5 | -0.43(-1.34,0.48) |  | -0.50(-1.76,0.77) |  | 0.60(-1.20,2.40) |  | -5.81(-9.50,-2.12) |  | -1.60(-4.55,1.35) |  | 0.67(-2.49,3.82) |
| 6-7 | -1.71(-2.18,-1.24) |  | -1.69(-2.42,-0.96) |  | -3.00(-3.81,-2.19) |  | -5.22(-6.37,-4.08) |  | -2.23(-3.95,-0.51) |  | -0.46(-2.30,1.39) |
| **Amount of tea consumption (g/d)** | | | | | | | | | | | |
| ≤2.0 | -1.62(-2.36,-0.88) |  | -2.11(-3.09,-1.12) |  | -4.62(-6.02,-3.22) |  | -4.78(-6.21,-3.35) |  | -3.51(-6.42,-0.59) |  | -1.79(-4.37,0.79) |
| 2.1-4.0 | -1.72(-2.38,-1.07) |  | -1.12(-2.17,-0.06) |  | -4.62(-5.92,-3.32) |  | -7.54(-9.45,-5.63) |  | -2.14(-4.70,0.43) |  | 0.46(-2.31,3.23) |
| 4.1-8.0 | -1.09(-1.82,-0.36) |  | -1.66(-2.85,-0.47) |  | -1.02(-2.18,0.14) |  | -7.89(-10.23,-5.56) |  | -1.56(-4.48,1.35) |  | 1.47(-1.61,4.56) |
| ≥8.1 | -1.23(-2.04,-0.43) |  | -0.67(-1.94,0.60) |  | -1.72(-2.71,-0.74) |  | -3.43(-5.09,-1.78) |  | -0.55(-3.04,1.95) |  | 0.20(-2.72,3.12) |
| **Outcome= DBP** | | | | | | | | | | | |
| **Duration of tea consumption (years)** | | | | | | | | | | | |
| ≤15 | 0.64(0.21,1.08) |  | 0.43(-0.21,1.07) |  | -0.78(-1.78,0.22) |  | 1.57(-0.54,3.68) |  | 0.27(-0.99,1.53) |  | 1.03(-0.26,2.33) |
| 16-40 | 0.38(0.05,0.71) |  | 0.23(-0.27,0.72) |  | -0.93(-1.52,-0.34) |  | -0.33(-1.18,0.52) |  | -0.07(-1.39,1.25) |  | 0.21(-1.29,1.71) |
| >40 | 0.45(-0.18,1.09) |  | -0.23(-1.26,0.80) |  | 0.38(-0.34,1.10) |  | 1.16(0.23,2.09) |  | -2.12(-4.97,0.73) |  | 0.85(-1.53,3.22) |
| **Frequency of tea consumption (**d/w**)** | | | | | | | | | | | |
| 1-2 | 0.49(-0.16,1.15) |  | -0.69(-1.65,0.28) |  | -0.64(-1.93,0.66) |  | -0.49(-3.29,2.30) |  | -0.43(-2.68,1.83) |  | -0.16(-2.36,2.03) |
| 3-5 | 0.64(0.06,1.23) |  | 0.67(-0.14,1.48) |  | -0.67(-1.83,0.49) |  | 0.23(-2.14,2.61) |  | 0.53(-1.37,2.42) |  | 0.73(-1.29,2.76) |
| 6-7 | 0.49(0.19,0.80) |  | 0.41(-0.06,0.88) |  | -0.53(-1.06,-0.01) |  | 0.41(-0.32,1.15) |  | -0.14(-1.24,0.97) |  | 0.79(-0.40,1.97) |
| **Amount of tea consumption (**g/d**)** | | | | | | | | | | | |
| ≤2.0 | 0.39(-0.09,0.86) |  | -0.10(-0.73,0.53) |  | 0.69(-0.21,1.59) |  | 1.67(0.75,2.59) |  | -0.71(-2.59,1.16) |  | 0.93(-0.72,2.59) |
| 2.1-4.0 | 0.22(-0.20,0.64) |  | 0.44(-0.24,1.11) |  | -0.94(-1.78,-0.10) |  | -0.75(-1.98,0.48) |  | -0.30(-1.94,1.35) |  | 1.15(-0.62,2.93) |
| 4.1-8.0 | 0.73(0.26,1.20) |  | 0.04(-0.72,0.81) |  | -0.43(-1.18,0.32) |  | -1.14(-2.64,0.37) |  | 0.69(-1.19,2.56) |  | 1.04(-0.94,3.02) |
| ≥8.1 | 0.62(0.10,1.13) |  | 0.82(0.01,1.64) |  | -0.90(-1.53,-0.26) |  | -0.16(-1.23,0.90) |  | 0.02(-1.58,1.63) |  | -0.46(-2.34,1.41) |
|  | Green tea |  | Scented tea |  | Dark tea |  | Sweet tea |  | Black tea |  | Other tea |
|  | β(95%CI) |  | β(95%CI) |  | β(95%CI) |  | β(95%CI) |  | β(95%CI) |  | β(95%CI) |
| **≥ 46.5 g/w** | | | | | | | | | | | |
| **Outcome = SBP** | | | | | | | | | | | |
| **Duration of tea consumption (years)** | | | | | | | | | | | |
| ≤15 | -0.25(-1.12,0.62) |  | -2.14(-3.61,-0.67) |  | -3.25(-5.76,-0.74) |  | -4.53(-8.88,-0.18) |  | -1.07(-4.19,2.05) |  | 1.05(-1.93,4.04) |
| 16-40 | -2.04(-2.72,-1.35) |  | -2.66(-3.75,-1.56) |  | -1.68(-3.07,-0.28) |  | -5.15(-7.25,-3.05) |  | -3.02(-6.16,0.12) |  | -1.07(-4.38,2.24) |
| >40 | -3.29(-4.50,-2.09) |  | -1.70(-3.91,0.51) |  | -4.00(-5.68,-2.31) |  | -4.23(-6.36,-2.10) |  | -3.71(-8.73,1.31) |  | -9.36(-15.43,-3.29) |
| **Frequency of tea consumption (d/w)** | | | | | | | | | | | |
| 1-2 | -1.81(-3.10,-0.52) |  | -3.86(-6.27,-1.45) |  | -4.01(-7.26,-0.76) |  | -3.16(-8.94,2.61) |  | -0.03(-4.64,4.58) |  | -2.43(-7.70,2.83) |
| 3-5 | -1.10(-2.33,0.13) |  | -2.67(-4.46,-0.87) |  | -3.58(-6.95,-0.20) |  | -6.39(-11.98,-0.81) |  | -3.20(-8.47,2.07) |  | -1.81(-7.15,3.54) |
| 6-7 | -1.84(-2.45,-1.22) |  | -2.10(-3.13,-1.08) |  | -2.39(-3.65,-1.14) |  | -4.70(-6.52,-2.88) |  | -2.72(-5.25,-0.20) |  | -0.64(-3.22,1.94) |
| **Amount of tea consumption (g/d)** | | | | | | | | | | | |
| ≤2.0 | -2.23(-3.23,-1.22) |  | -2.47(-3.87,-1.08) |  | -5.65(-7.48,-3.82) |  | -4.87(-6.84,-2.89) |  | -0.68(-5.19,3.83) |  | -4.96(-8.84,-1.07) |
| 2.1-4.0 | -1.58(-2.44,-0.72) |  | -2.46(-3.90,-1.02) |  | -4.45(-6.38,-2.52) |  | -5.77(-8.50,-3.04) |  | -3.95(-7.46,-0.43) |  | -0.94(-4.88,3.00) |
| 4.1-8.0 | -1.69(-2.63,-0.75) |  | -3.04(-4.77,-1.31) |  | 1.54(-0.19,3.26) |  | -3.08(-6.85,0.68) |  | -5.31(-9.82,-0.80) |  | 2.21(-2.46,6.88) |
| ≥8.1 | -1.16(-2.22,-0.10) |  | -0.93(-3.02,1.16) |  | -2.49(-4.21,-0.78) |  | -2.99(-6.51,0.54) |  | 0.35(-3.66,4.37) |  | 0.93(-3.40,5.26) |
| **Outcome= DBP** | | | | | | | | | | | |
| **Duration of tea consumption (years)** | | | | | | | | | | | |
| ≤15 | 0.42(-0.13,0.97) |  | 0.08(-0.85,1.00) |  | -0.70(-2.28,0.88) |  | 1.37(-1.38,4.12) |  | -1.50(-3.47,0.46) |  | -0.51(-2.39,1.37) |
| 16-40 | 0.20(-0.23,0.63) |  | -0.23(-0.93,0.46) |  | -0.99(-1.87,-0.11) |  | -0.79(-2.11,0.54) |  | -1.55(-3.53,0.43) |  | 0.01(-2.07,2.10) |
| >40 | 0.09(-0.67,0.85) |  | -0.05(-1.44,1.35) |  | 0.51(-0.55,1.58) |  | 2.43(1.08,3.77) |  | 1.29(-1.87,4.45) |  | -2.30(-6.12,1.53) |
| **Frequency of tea consumption (**d/w**)** | | | | | | | | | | | |
| 1-2 | -0.58(-1.40,0.23) |  | -1.27(-2.79,0.25) |  | 0.11(-1.94,2.16) |  | 0.52(-3.13,4.18) |  | -2.47(-5.38,0.43) |  | -1.93(-5.24,1.38) |
| 3-5 | 0.36(-0.42,1.13) |  | -0.76(-1.90,0.37) |  | -2.20(-4.33,-0.07) |  | -1.69(-5.22,1.84) |  | -1.75(-5.08,1.57) |  | 0.06(-3.30,3.42) |
| 6-7 | 0.37(-0.02,0.76) |  | 0.29(-0.36,0.94) |  | -0.35(-1.14,0.44) |  | 0.92(-0.23,2.07) |  | -0.49(-2.08,1.10) |  | -0.35(-1.97,1.28) |
| **Amount of tea consumption (**g/d**)** | | | | | | | | | | | |
| ≤2.0 | 0.22(-0.41,0.85) |  | 0.27(-0.61,1.15) |  | -0.16(-1.32,1.00) |  | 1.19(-0.06,2.44) |  | -2.27(-5.11,0.57) |  | -0.88(-3.33,1.56) |
| 2.1-4.0 | -0.08(-0.62,0.46) |  | -0.41(-1.31,0.50) |  | -1.19(-2.41,0.04) |  | -0.68(-2.40,1.05) |  | -1.37(-3.59,0.84) |  | -1.28(-3.76,1.20) |
| 4.1-8.0 | 0.53(-0.07,1.12) |  | -0.26(-1.35,0.83) |  | 0.05(-1.04,1.14) |  | 1.35(-1.03,3.73) |  | -1.31(-4.15,1.53) |  | 1.19(-1.75,4.12) |
| ≥8.1 | 0.36(-0.31,1.02) |  | -0.10(-1.42,1.22) |  | -0.83(-1.92,0.25) |  | 1.06(-1.17,3.29) |  | 0.84(-1.69,3.37) |  | -0.64(-3.36,2.08) |

Note: Adjusted for age, sex, ethnicity, marital status, education, occupation, family income, BMI, MET, smoking status, alcohol use status, salt intake, vegetable intake, fruits intake, dairy intake, family history of hypertension, and physician-diagnosed diseases. βindicates unstandardized partial regression coefficient.

Table S3 Relationship between variables of tea consumption habits and SBP, DBP in participants with hypertensive BP and physician-diagnosed cardiovascular disease

|  | Green tea |  | Scented tea |  | Dark tea |  | Sweet tea |  | Black tea |  | Other tea |
| --- | --- | --- | --- | --- | --- | --- | --- | --- | --- | --- | --- |
|  | β(95%CI) |  | β(95%CI) |  | β(95%CI) |  | β(95%CI) |  | β(95%CI) |  | β(95%CI) |
| **Outcome = SBP** | | | | | | | | | | | |
| **Duration of tea consumption (years)** | | | | | | | | | | | |
| ≤15 | -0.21(-0.74,0.32) |  | -1.37(-2.19,-0.55) |  | -2.75(-4.07,-1.44) |  | -5.67(-8.24,-3.11) |  | -0.53(-2.18,1.12) |  | 1.71(0.04,3.38) |
| 16-40 | -1.88(-2.29,-1.47) |  | -1.88(-2.50,-1.25) |  | -1.70(-2.46,-0.94) |  | -5.00(-6.11,-3.89) |  | -2.75(-4.46,-1.05) |  | -0.96(-2.85,0.94) |
| >40 | -3.03(-3.79,-2.28) |  | -2.41(-3.69,-1.14) |  | -4.18(-5.09,-3.26) |  | -5.67(-6.84,-4.50) |  | -5.78(-8.98,-2.57) |  | -4.74(-7.82,-1.67) |
| **Frequency of tea consumption (d/w)** | | | | | | | | | | | |
| 1-2 | -1.31(-2.11,-0.52) |  | -2.47(-3.75,-1.20) |  | -2.52(-4.23,-0.81) |  | -5.26(-8.71,-1.81) |  | -0.57(-3.38,2.23) |  | -0.33(-3.17,2.51) |
| 3-5 | -0.67(-1.40,0.06) |  | -1.33(-2.36,-0.30) |  | -0.32(-1.92,1.27) |  | -6.01(-9.06,-2.97) |  | -1.97(-4.52,0.58) |  | -0.06(-2.77,2.65) |
| 6-7 | -1.77(-2.15,-1.40) |  | -1.79(-2.38,-1.20) |  | -2.88(-3.56,-2.20) |  | -5.30(-6.26,-4.34) |  | -2.52(-3.92,-1.12) |  | -0.41(-1.90,1.08) |
| **Amount of tea consumption (g/d)** | | | | | | | | | | | |
| ≤2.0 | -1.81(-2.40,-1.21) |  | -2.24(-3.04,-1.44) |  | -5.25(-6.35,-4.16) |  | -5.13(-6.26,-4.00) |  | -2.66(-5.08,-0.23) |  | -2.93(-5.05,-0.81) |
| 2.1-4.0 | -1.68(-2.20,-1.16) |  | -1.58(-2.42,-0.74) |  | -4.59(-5.66,-3.52) |  | -7.05(-8.59,-5.51) |  | -3.24(-5.26,-1.23) |  | 0.07(-2.19,2.33) |
| 4.1-8.0 | -1.36(-1.93,-0.78) |  | -2.21(-3.19,-1.23) |  | -0.36(-1.32,0.60) |  | -6.74(-8.68,-4.80) |  | -2.61(-5.04,-0.17) |  | 2.17(-0.38,4.72) |
| ≥8.1 | -1.22(-1.85,-0.58) |  | -0.65(-1.72,0.43) |  | -1.73(-2.58,-0.88) |  | -3.51(-5.00,-2.03) |  | -0.14(-2.26,1.98) |  | 0.72(-1.71,3.14) |
| **Outcome= DBP** | | | | | | | | | | | |
| **Duration of tea consumption (years)** | | | | | | | | | | | |
| ≤15 | 0.58(0.24,0.92) |  | 0.27(-0.25,0.79) |  | -0.75(-1.59,0.09) |  | 1.05(-0.59,2.68) |  | -0.30(-1.36,0.75) |  | 0.55(-0.51,1.61) |
| 16-40 | 0.32(0.06,0.58) |  | 0.07(-0.33,0.46) |  | -0.94(-1.42,-0.45) |  | -0.54(-1.25,0.16) |  | -0.54(-1.62,0.54) |  | 0.26(-0.94,1.47) |
| >40 | 0.31(-0.17,0.79) |  | -0.26(-1.07,0.56) |  | 0.43(-0.15,1.02) |  | 1.49(0.74,2.24) |  | -0.23(-2.27,1.81) |  | 0.02(-1.94,1.97) |
| **Frequency of tea consumption (**d/w**)** | | | | | | | | | | | |
| 1-2 | 0.10(-0.40,0.61) |  | -0.88(-1.69,-0.06) |  | -0.52(-1.61,0.57) |  | -0.26(-2.47,1.94) |  | -1.18(-2.96,0.60) |  | -0.36(-2.17,1.44) |
| 3-5 | 0.60(0.14,1.06) |  | 0.12(-0.53,0.78) |  | -1.06(-2.08,-0.05) |  | -0.48(-2.42,1.46) |  | -0.19(-1.82,1.43) |  | 0.57(-1.15,2.29) |
| 6-7 | 0.44(0.21,0.68) |  | 0.36(-0.02,0.74) |  | -0.45(-0.89,-0.02) |  | 0.48(-0.13,1.09) |  | -0.20(-1.09,0.69) |  | 0.41(-0.53,1.36) |
| **Amount of tea consumption (**g/d**)** | | | | | | | | | | | |
| ≤2.0 | 0.35(-0.03,0.72) |  | 0.01(-0.50,0.52) |  | 0.28(-0.42,0.97) |  | 1.39(0.67,2.11) |  | -1.21(-2.75,0.33) |  | 0.26(-1.09,1.61) |
| 2.1-4.0 | 0.11(-0.22,0.44) |  | 0.09(-0.45,0.62) |  | -0.98(-1.67,-0.29) |  | -0.83(-1.81,0.15) |  | -0.57(-1.86,0.71) |  | 0.41(-1.03,1.85) |
| 4.1-8.0 | 0.68(0.32,1.05) |  | -0.12(-0.75,0.50) |  | -0.31(-0.92,0.30) |  | -0.49(-1.73,0.75) |  | 0.09(-1.46,1.64) |  | 1.25(-0.37,2.87) |
| ≥8.1 | 0.51(0.10,0.91) |  | 0.59(-0.10,1.27) |  | -0.81(-1.35,-0.27) |  | -0.15(-1.09,0.80) |  | 0.21(-1.14,1.56) |  | -0.42(-1.96,1.12) |

Note: Adjusted for age, sex, ethnicity, marital status, education, occupation, family income, BMI, MET, smoking status, alcohol use status, salt intake, vegetable intake, fruits intake, dairy intake, family history of hypertension, and physician-diagnosed diseases. βindicates unstandardized partial regression coefficient.

Table S4 Relationship between variables of tea consumption habits and SBP, DBP in participants with hypertensive BP, physician-diagnosed hypertension and cardiovascular disease

|  | Green tea |  | Scented tea |  | Dark tea |  | Sweet tea |  | Black tea |  | Other tea |
| --- | --- | --- | --- | --- | --- | --- | --- | --- | --- | --- | --- |
|  | β(95%CI) |  | β(95%CI) |  | β(95%CI) |  | β(95%CI) |  | β(95%CI) |  | β(95%CI) |
| **Outcome = SBP** | | | | | | | | | | | |
| **Duration of tea consumption (years)** | | | | | | | | | | | |
| ≤15 | -0.26(-0.82,0.29) |  | -1.02(-1.87,-0.17) |  | -2.91(-4.29,-1.53) |  | -5.10(-7.74,-2.45) |  | 0.23(-1.53,1.99) |  | 1.28(-0.42,2.98) |
| 16-40 | -1.66(-2.08,-1.24) |  | -2.07(-2.71,-1.43) |  | -1.89(-2.69,-1.10) |  | -4.84(-6.01,-3.68) |  | -3.23(-5.04,-1.43) |  | -0.43(-2.31,1.44) |
| >40 | -3.23(-3.94,-2.53) |  | -2.91(-4.07,-1.74) |  | -3.68(-4.59,-2.76) |  | -4.36(-5.52,-3.19) |  | -4.99(-8.08,-1.91) |  | -2.45(-5.25,0.35) |
| **Frequency of tea consumption (d/w)** | | | | | | | | | | | |
| 1-2 | -1.26(-2.08,-0.44) |  | -2.20(-3.52,-0.88) |  | -2.32(-4.12,-0.52) |  | -4.29(-7.66,-0.92) |  | -0.32(-3.37,2.74) |  | 0.38(-2.52,3.27) |
| 3-5 | -0.87(-1.62,-0.11) |  | -1.43(-2.51,-0.34) |  | -0.72(-2.39,0.96) |  | -6.12(-9.22,-3.02) |  | -1.12(-3.82,1.58) |  | -0.56(-3.31,2.19) |
| 6-7 | -1.76(-2.14,-1.39) |  | -1.87(-2.46,-1.28) |  | -2.90(-3.59,-2.20) |  | -4.56(-5.54,-3.57) |  | -2.75(-4.21,-1.30) |  | 0.01(-1.44,1.46) |
| **Amount of tea consumption (g/d)** | | | | | | | | | | | |
| ≤2.0 | -2.04(-2.65,-1.44) |  | -2.47(-3.28,-1.67) |  | -5.09(-6.21,-3.98) |  | -4.63(-5.79,-3.48) |  | -1.56(-4.11,0.99) |  | -2.84(-4.90,-0.77) |
| 2.1-4.0 | -1.56(-2.08,-1.04) |  | -1.72(-2.57,-0.87) |  | -4.23(-5.33,-3.13) |  | -5.89(-7.46,-4.33) |  | -3.49(-5.61,-1.37) |  | -0.25(-2.49,1.98) |
| 4.1-8.0 | -1.37(-1.95,-0.78) |  | -1.85(-2.84,-0.87) |  | -0.82(-1.82,0.17) |  | -6.49(-8.47,-4.52) |  | -2.44(-5.00,0.12) |  | 2.94(0.40,5.47) |
| ≥8.1 | -1.13(-1.77,-0.49) |  | -0.94(-2.03,0.16) |  | -1.74(-2.62,-0.86) |  | -2.56(-4.08,-1.05) |  | -0.65(-2.87,1.56) |  | 1.46(-0.97,3.88) |
| **Outcome= DBP** | | | | | | | | | | | |
| **Duration of tea consumption (years)** | | | | | | | | | | | |
| ≤15 | 0.54(0.20,0.88) |  | 0.31(-0.21,0.84) |  | -0.66(-1.51,0.19) |  | 1.31(-0.32,2.95) |  | 0.06(-1.02,1.15) |  | 0.14(-0.90,1.19) |
| 16-40 | 0.48(0.22,0.74) |  | -0.09(-0.49,0.30) |  | -1.18(-1.68,-0.69) |  | -0.59(-1.31,0.13) |  | -0.87(-1.98,0.24) |  | 0.66(-0.49,1.81) |
| >40 | 0.28(-0.16,0.72) |  | -0.43(-1.15,0.29) |  | 0.84(0.27,1.40) |  | 2.33(1.61,3.05) |  | -0.65(-2.55,1.25) |  | 1.75(0.03,3.47) |
| **Frequency of tea consumption (**d/w**)** | | | | | | | | | | | |
| 1-2 | 0.03(-0.48,0.54) |  | -0.77(-1.58,0.05) |  | -0.34(-1.45,0.77) |  | 0.45(-1.63,2.53) |  | -1.11(-2.99,0.76) |  | 0.15(-1.63,1.93) |
| 3-5 | 0.54(0.08,1.00) |  | 0.12(-0.55,0.79) |  | -1.19(-2.23,-0.15) |  | -0.73(-2.65,1.18) |  | 0.01(-1.65,1.67) |  | 0.55(-1.14,2.24) |
| 6-7 | 0.53(0.29,0.76) |  | 0.18(-0.19,0.54) |  | -0.42(-0.85,0.01) |  | 1.00(0.39,1.61) |  | -0.43(-1.33,0.47) |  | 0.67(-0.22,1.56) |
| **Amount of tea consumption (**g/d**)** | | | | | | | | | | | |
| ≤2.0 | 0.26(-0.11,0.63) |  | -0.24(-0.74,0.25) |  | 0.49(-0.20,1.18) |  | 1.70(0.99,2.41) |  | -0.67(-2.24,0.90) |  | 0.57(-0.71,1.84) |
| 2.1-4.0 | 0.20(-0.12,0.53) |  | -0.15(-0.67,0.38) |  | -0.64(-1.32,0.04) |  | -0.18(-1.14,0.79) |  | -0.95(-2.26,0.35) |  | 0.80(-0.58,2.17) |
| 4.1-8.0 | 0.76(0.40,1.12) |  | 0.04(-0.57,0.64) |  | -0.62(-1.24,-0.01) |  | -0.26(-1.48,0.96) |  | 0.11(-1.46,1.69) |  | 1.01(-0.55,2.57) |
| ≥8.1 | 0.69(0.29,1.08) |  | 0.58(-0.10,1.25) |  | -0.73(-1.27,-0.19) |  | 0.68(-0.25,1.62) |  | -0.02(-1.38,1.34) |  | 0.02(-1.47,1.52) |

Note: Adjusted for age, sex, ethnicity, marital status, education, occupation, family income, BMI, MET, smoking status, alcohol use status, salt intake, vegetable intake, fruits intake, dairy intake, family history of hypertension, and physician-diagnosed diseases. βindicates unstandardized partial regression coefficient.


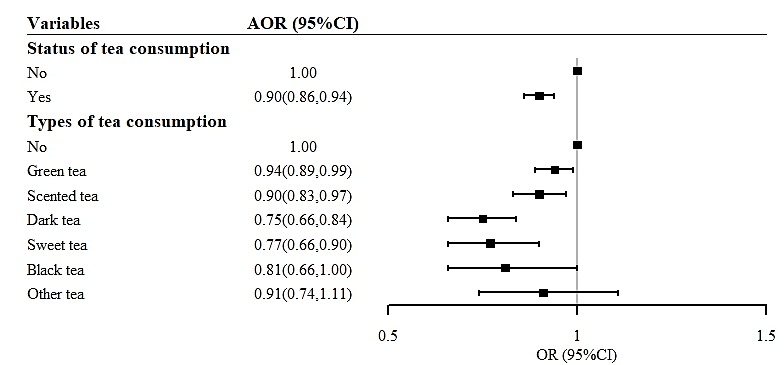


Fig S1. Relationship between types of tea consumption and hypertensive BP in participants with hypertensive BP and physician-diagnosed cardiovascular disease

Note: AORs (95%CIs) were adjusted for age, sex, ethnicity, marital status, education, occupation, family income, BMI, MET, smoking status, alcohol use status, salt intake, vegetable intake, fruits intake, dairy intake, family history of hypertension, and physician-diagnosed diseases.


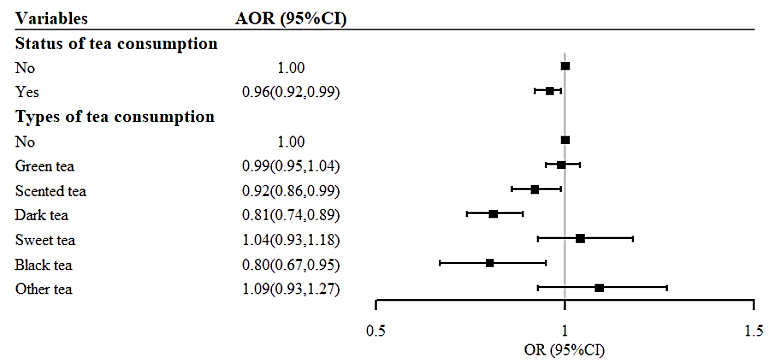


Fig S2. Relationship between types of tea consumption and hypertensive BP in participants with hypertensive BP, physician-diagnosed hypertension and cardiovascular disease

Note: AORs (95%CIs) were adjusted for age, sex, ethnicity, marital status, education, occupation, family income, BMI, MET, smoking status, alcohol use status, salt intake, vegetable intake, fruits intake, dairy intake, family history of hypertension, and physician-diagnosed diseases.


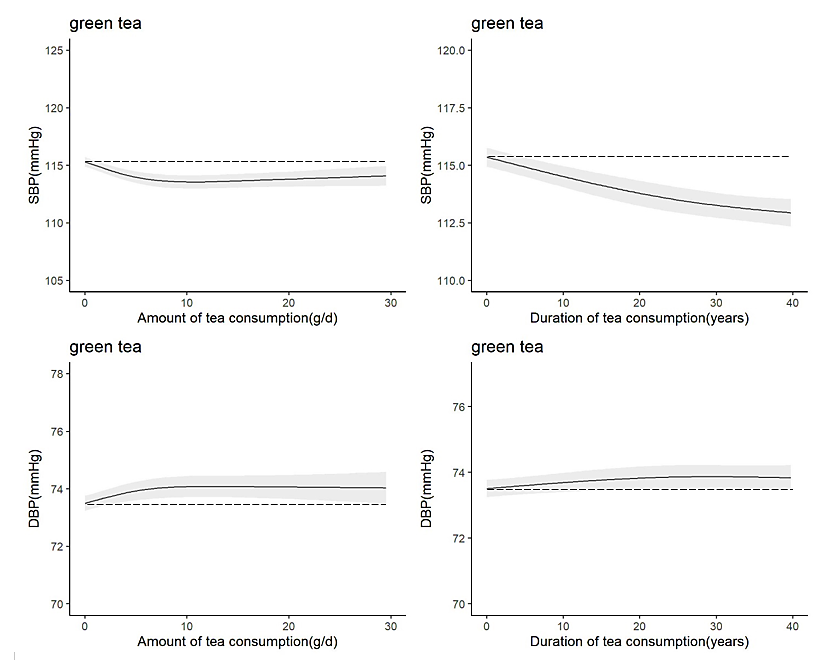


Fig S3. Relationships between variables of green tea consumption and SBP, DBP

Note: model adjusted for age, sex, ethnicity, marital status, education, occupation, family income, BMI, MET, smoking status, alcohol use status, salt intake, vegetable intake, fruits intake, dairy intake, family history of hypertension, and physician-diagnosed diseases.


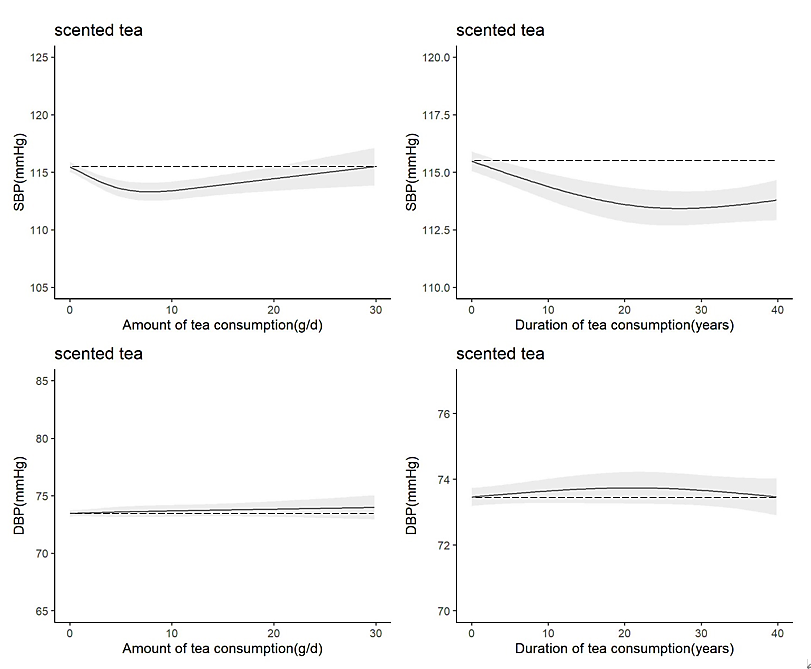


Fig S4. Relationships between variables of scented tea consumption and SBP, DBP

Note: model adjusted for age, sex, ethnicity, marital status, education, occupation, family income, BMI, MET, smoking status, alcohol use status, salt intake, vegetable intake, fruits intake, dairy intake, family history of hypertension, and physician-diagnosed diseases.


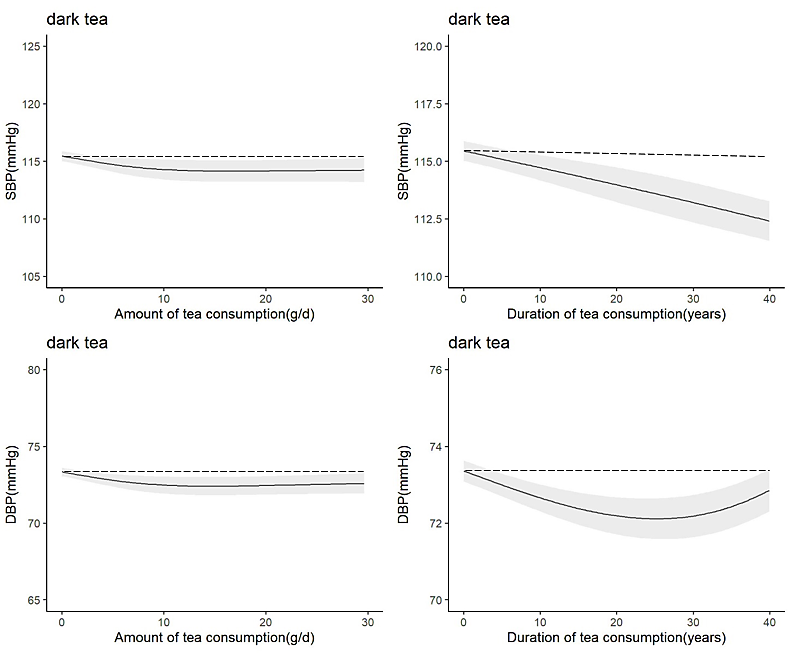


Fig S5. Relationships between variables of dark tea consumption and SBP, DBP

Note: model adjusted for age, sex, ethnicity, marital status, education, occupation, family income, BMI, MET, smoking status, alcohol use status, salt intake, vegetable intake, fruits intake, dairy intake, family history of hypertension, and physician-diagnosed diseases.


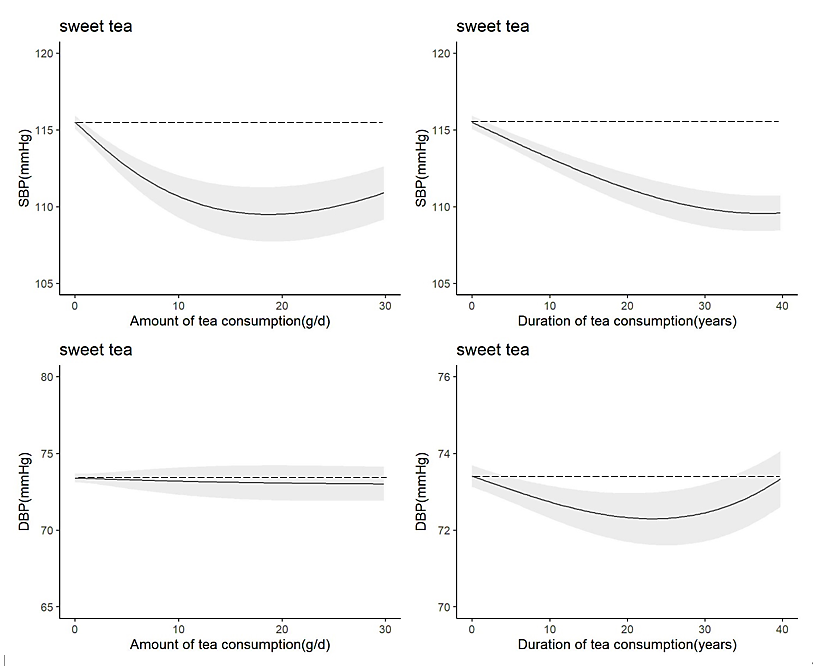


Fig S6. Relationships between variables of sweet tea consumption and SBP, DBP

Note: model adjusted for age, sex, ethnicity, marital status, education, occupation, family income, BMI, MET, smoking status, alcohol use status, salt intake, vegetable intake, fruits intake, dairy intake, family history of hypertension, and physician-diagnosed diseases.


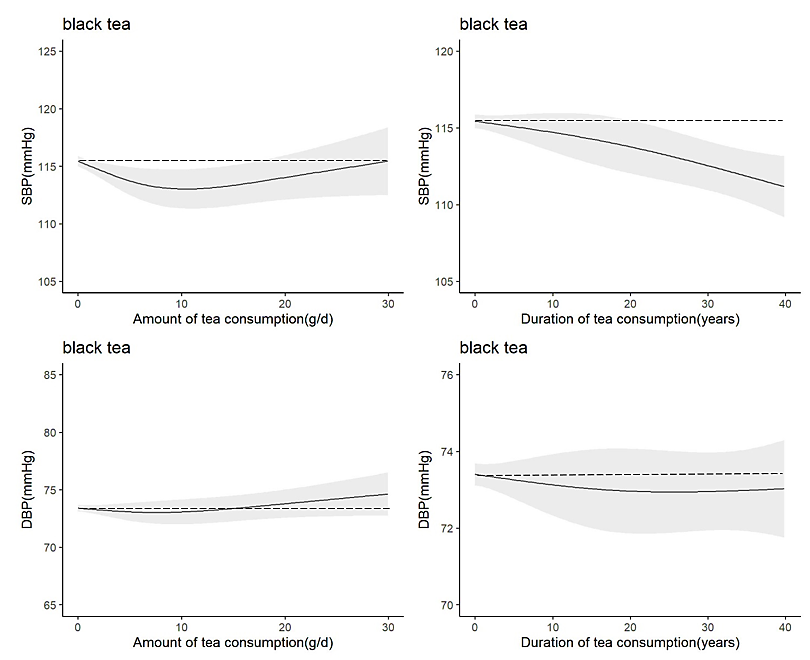


Fig S7. Relationship between variables of scented tea consumption and SBP, DBP

Note: model adjusted for age, sex, ethnicity, marital status, education, occupation, family income, BMI, MET, smoking status, alcohol use status, salt intake, vegetable intake, fruits intake, dairy intake, family history of hypertension, and physician-diagnosed diseases.
